# Polyamidoamine Nanoparticles for the Oral Administration of Antimalarial Drugs

**DOI:** 10.3390/pharmaceutics10040225

**Published:** 2018-11-10

**Authors:** Elisabet Martí Coma-Cros, Arnau Biosca, Joana Marques, Laura Carol, Patricia Urbán, Diana Berenguer, Maria Cristina Riera, Michael Delves, Robert E. Sinden, Juan José Valle-Delgado, Lefteris Spanos, Inga Siden-Kiamos, Paula Pérez, Krijn Paaijmans, Matthias Rottmann, Amedea Manfredi, Paolo Ferruti, Elisabetta Ranucci, Xavier Fernàndez-Busquets

**Affiliations:** 1 Barcelona Institute for Global Health (ISGlobal, Hospital Clínic-Universitat de Barcelona), Rosselló 149-153, ES-08036 Barcelona, Spain; elisabet.marti@isglobal.org (E.M.C.-C.); abiosca@ibecbarcelona.eu (A.B.); joana.a.marques@gmail.com (J.M.); lauracarol.20@gmail.com (L.C.); urban.patricia@gmail.com (P.U.); paupga20@gmail.com (P.P.); krijn.paaijmans@asu.edu (K.P.); 2 Nanomalaria Group, Institute for Bioengineering of Catalonia (IBEC), The Barcelona Institute of Science and Technology, Baldiri Reixac 10-12, ES-08028 Barcelona, Spain; 3 Laboratori de Parasitologia, Departament de Microbiologia i Parasitologia Sanitàries, Facultat de Farmàcia, Universitat de Barcelona, Av. Joan XXIII s/n, ES-08028 Barcelona, Spain; berenguer.diana@gmail.com (D.B.); mcriera@ub.edu (M.C.R.); 4 Department of Life Sciences, Imperial College, South Kensington, London SW7 2AZ, UK; michael.delves@lshtm.ac.uk (M.D.); r.sinden@imperial.ac.uk (R.E.S.); 5 Department of Bioproducts and Biosystems, School of Chemical Engineering, Aalto University, P.O. Box 16300, FI-00076 Aalto, Finland; juanjose.valledelgado@aalto.fi; 6 Institute of Molecular Biology and Biotechnology, FORTH, N. Plastira 100, 700 13 Heraklion, Greece; spanos@imbb.forth.gr (L.S.); inga@imbb.forth.gr (I.S.-K.); 7 Swiss Tropical and Public Health Institute, Socinstrasse 57, CH-4051 Basel, Switzerland; matthias.rottmann@swisstph.ch; 8 Universität Basel, Petersplatz 1, CH-4003 Basel, Switzerland; 9 Dipartimento di Chimica, Università degli Studi di Milano, via Golgi 19, IT-20133 Milano, Italy; amedea.manfredi@unimi.it (A.M.); paolo.ferruti@unimi.it (P.F.); elisabetta.ranucci@unimi.it (E.R.)

**Keywords:** *Anopheles*, antimalarial drugs, malaria, mosquitoes, nanomedicine, nanotechnology, *Plasmodium*, polymers, polyamidoamines, targeted drug delivery

## Abstract

Current strategies for the mass administration of antimalarial drugs demand oral formulations to target the asexual *Plasmodium* stages in the peripheral bloodstream, whereas recommendations for future interventions stress the importance of also targeting the transmission stages of the parasite as it passes between humans and mosquitoes. Orally administered polyamidoamine (PAA) nanoparticles conjugated to chloroquine reached the blood circulation and cured *Plasmodium yoelii*-infected mice, slightly improving the activity of the free drug and inducing in the animals immunity against malaria. Liquid chromatography with tandem mass spectrometry analysis of affinity chromatography-purified PAA ligands suggested a high adhesiveness of PAAs to *Plasmodium falciparum* proteins, which might be the mechanism responsible for the preferential binding of PAAs to *Plasmodium*-infected erythrocytes vs. non-infected red blood cells. The weak antimalarial activity of some PAAs was found to operate through inhibition of parasite invasion, whereas the observed polymer intake by macrophages indicated a potential of PAAs for the treatment of certain coinfections such as *Plasmodium* and *Leishmania*. When fluorescein-labeled PAAs were fed to females of the malaria mosquito vectors *Anopheles atroparvus* and *Anopheles gambiae*, persistent fluorescence was observed in the midgut and in other insect’s tissues. These results present PAAs as a versatile platform for the encapsulation of orally administered antimalarial drugs and for direct administration of antimalarials to mosquitoes, targeting mosquito stages of *Plasmodium*.

## 1. Introduction

Oral administration of antimalarial drugs is the best route in terms of patient compliance: it makes people feel less sick and reduces medical cost because of its ease of administration [1]. Currently used antimalarials, however, are not easily dissolved in aqueous solvents or show poor membrane permeability, which, in addition to their sensitivity to degradation, makes their oral delivery challenging [2]. To overcome this obstacle, incorporating polymers in the formulation is a strategy that has been used to give drugs increased solubility and membrane-trespassing capacity, and to protect labile compounds from digestive enzymes and extreme pH variations [3]. Polymeric nanocarriers are generally stable during gastrointestinal tract (GIT) transit [4], and given the versatility associated with their constituent materials (natural, semisynthetic or synthetic) as well as the variety of methods by which they are processed (e.g., spray-drying, emulsion techniques, precipitation, solvent extraction/evaporation), they can be used for the delivery of a wide range of cargoes [3]. Furthermore, advances in polymer chemistry enable exquisite control over the nanoarchitecture and biophysical properties of polymeric nanocarriers, which facilitates controlled or triggered drug delivery [5]. Polycations, in particular, are especially adept at oral administration because of their capacity to promote drug absorption by a variety of mechanisms, among which an increased GIT retention due to their mucoadhesive properties [6]. The systemic exposure to orally delivered polycations is low compared to the parenteral route, and therefore better tolerance is expected. Certain polycations such as polyamidoamine (PAA) polymers have the capacity to increase the epithelial permeability to conjugated drugs and biomacromolecules [6], mainly by potentiating paracellular transport although some systems may boost transcytotic passage as well. Also, anionic PAA polymers have been described as more efficiently transported across the intestinal epithelium, primarily through the paracellular route [7]. However, despite numerous studies on oral PAA bioavailability [6,7,8,9,10], it remains unexplored to what extent this administration method provides the necessary plasma drug concentrations for the pharmaceutical development of oral antimalarial formulations targeting *Plasmodium* parasites inside red blood cells (RBCs).

The PAA structures AGMA1 and ISA23, with respective isoelectric points of 10.0 and 5.5, had been explored for the encapsulation and targeted delivery of the antimalarial drugs chloroquine (CQ) and primaquine [11]. Fluorescence-assisted cell sorting, confocal fluorescence microscopy, and transmission electron microscopy results indicated that AGMA1 and ISA23 polymers with hydrodynamic radii around 7 nm had specific targeting to *Plasmodium*-infected RBCs (pRBCs) vs. non-infected erythrocytes, and to intraerythrocytic *Plasmodium falciparum* and *Plasmodium yoelii* merozoites, whereas AGMA1 itself exhibited a mild antimalarial activity. Mice inoculated with a lethal strain of the murine malaria species *P. yoelii* were freed of parasites after intraperitoneal administration of PAA-encapsulated CQ, whereas the same amount of free drug was unable to cure the animals.

Notwithstanding these promising results, several questions remained open, among them which is the mechanism behind the specificity of binding of PAAs to pRBCs, and whether PAAs could be applied to the current need of mass drug administration for non-complicated malaria by oral delivery [1]. With these two main objectives, previous synthetic protocols [11] have been adapted to obtain larger PAA structures that will take longer to degrade in the aggressive GIT environment. We present the characterization of these new polymers in terms of structure, cellular and subcellular targeting, and antimalarial activity after CQ encapsulation, both in in vitro growth inhibition assays of the human malaria parasite *P. falciparum* and in in vivo experiments where *P. yoelii*-infected mice have been orally administered CQ-loaded PAAs. We also communicate the preliminary results of a radically new approach to malaria control and elimination, where PAAs were orally fed to the malaria mosquito vectors *Anopheles atroparvus* and *Anopheles gambiae* in preparation for future strategies that will target *Plasmodium* stages in the insect [12].

## 2. Materials and Methods

### 2.1. Polyamidoamine (PAA) Synthesis and Characterization

Unless otherwise indicated, all reagents were purchased from Sigma-Aldrich (St. Louis, MO, USA). Linear ISA23 [13], ISA1 [14], AGMA1 [15] and ARGO7 [16] were synthesized by stepwise Michael-type addition of *prim*- or *sec*-amines to bisacrylamides, as previously described [11], extending the reaction time to 14 days. Previously reported methods were followed for fluorescein isothiocyanate (FITC) labeling of the polymers [11,15] and for their loading with CQ [11]. FITC-labeled PAAs were obtained by reaction of FITC with PAAs bearing limited amounts of NH_2_ pendants, which were synthesized as previously reported for AGMA1 [15]. Therefore, fluorescein is bound to PAAs through a urethan function, which has proved to be extremely stable in physiological conditions [17]. The conjugation of PAAs with FITC was finally confirmed by NMR. To assess the encapsulated CQ content just before in vitro and in vivo analyses, PAAs were hydrolyzed overnight with 0.1 M NaOH, pH was neutralized with 5 M HCl to solubilize the precipitated drug, and CQ was determined by measuring A_340 nm_; a calibration curve was prepared with known concentrations of CQ diphosphate identically processed. The incorporation of sulfhydryl groups in PAAs was done using standard amino-functional pyridyl disulfide chemistry [18,19], which is described in detail in the Supplementary Materials for the three PAAs later used for in vivo assays. The nanoparticle size was assessed by nanoparticle tracking analysis, using a NanoSight LM10 (Malvern Instruments Ltd., Malvern, UK). The experiments were performed in phosphate-buffered saline (PBS) at 25 °C at a concentration of 10 mg/mL, except for ARGO7 which was dissolved at 2.5 mg/mL. For scanning electron microscopy (SEM) analysis, PAAs were dissolved in isopropanol at 5 µg/mL. Fifteen microliters of this PAA solution were placed on a Si sheet (MicroChemicals, Ulm, Germany) previously cleaned, first with acetone, then with ethanol, and finally with isopropanol. Samples were allowed to dry for 48 h at room temperature (RT, ca. 20 °C) and imaged with a Nova NanoSEM™ 230 scanning electron microscope (FEI Company, Eindhoven, The Netherlands). For atomic force microscopy (AFM) analysis, 10 μL of 1 mg/mL polymer solutions in double-deionized water (ddH_2_O; MilliQ system, Millipore, Burlington, MA, USA) were deposited on cleaved mica substrates and, after an adsorption time of about 5 min, 40–60 μL of ddH_2_O were added. High-resolution images were obtained with a MultiMode 8 atomic force microscope equipped with a NanoScope V controller (Bruker Corporation, Billerica, MA, USA) operating in ScanAsyst mode in liquid. ScanAsyst-Fluid+ probes (Bruker Corporation) were used in the experiments.

### 2.2. Matrix-Assisted Laser Desorption/Ionization Tandem Time-of-Flight (MALDI TOF/TOF) Analysis

PAA samples (1 mg/mL) were incubated for the times indicated in simulated fasted-state gastric fluid (80 μM Na-taurocholate, 34.2 mM NaCl, adjusted with HCl to pH 1.6). MALDI mass spectra were recorded using a 4800 Plus MALDI TOF/TOF (ABSciex, Framingham, MA, USA) instrument equipped with Nd:YAG solid state laser (355 nm, 200 Hz frequency, 3–7 ns pulse). Analyses were performed in both positive linear and reflector mode. Solutions of the samples (1 μL) were mixed with 1 μL of a saturated matrix solution of 2,5-dihydroxybenzoic acid in acetonitrile; 1 μL of this mixture was spotted on the sample plate and left to dry before the analysis.

### 2.3. Plasmodium falciparum Cell Culture and Parasite Growth Inhibition, Hemolysis and Unspecific Cytotoxicity Assays

*P. falciparum* 3D7 in vitro cultures were prepared as described elsewhere [20,21]. Parasites were grown at 37 °C in T25 flasks (SPL Life Sciences, Gyeonggi-do, Korea) containing human RBCs of blood group type B in a Roswell Park Memorial Institute (RPMI) medium supplemented with 5 g/L Albumax II and 2 mM glutamine (complete RPMI), following previously established conditions [22]. Two hundred microliters of these *Plasmodium* cultures were plated in 96-well plates and incubated for 48 h at 37 °C in the presence of free drugs and PAA-drug conjugates dissolved in RPMI. Parasitemia was determined by microscopic counting of blood smears or by flow cytometry as previously described [20].

The human blood used in this work was from voluntary donors and commercially obtained from the *Banc de Sang i Teixits* (www.bancsang.net). Blood was not collected specifically for this research; the purchased units had been discarded for transfusion, usually because of an excess of blood relative to anticoagulant solution. Prior to their use, blood units underwent the analytical checks specified in the current legislation. Before being delivered to us, unit data were anonymized and irreversibly dissociated, and any identification tag or label had been removed in order to guarantee the non-identification of the blood donor. No blood data were or will be supplied, in accordance with the current *Ley Orgánica de Protección de Datos* and *Ley de Investigación Biomédica*. The blood samples will not be used for studies other than those made explicit in this research.

Merozoite invasion inhibition assays were performed as previously described [23], in 150 μL of culture treated with PAAs dissolved in RPMI. The following equations were used to assess invasion and maturation rates:

(1) $$\mathrm{Invasion}=\frac{\mathrm{rings}\;\mathrm{day}\;n}{(\mathrm{trophozoites}+\mathrm{schizonts})\;\mathrm{day}\;n-1}$$

(2) $$\mathrm{Maturation}=\frac{(\mathrm{trophozoites}+\mathrm{schizonts})\;\mathrm{day}\;n}{\mathrm{rings}\;\mathrm{day}\;n-1}$$

Hemolysis and unspecific cytotoxicity assays were done as described elsewhere [20]. For AGMA1, cytotoxicity assays were done in Phenol Red-free medium.

### 2.4. Cell Targeting Analysis

Living *P. falciparum* 3D7 cultures were stained for 30 min with 4 μg/mL of the DNA dye Hoechst 33342 (λex/em: 350/461 nm). After three washes with complete or incomplete RPMI the cells were incubated with gentle stirring in the presence of 0.25 mg/mL PAA-FITC for 90 min in complete or incomplete RPMI at 37 °C. After washing with PBS, nonfixed samples were placed in an eight-well chamber slide system (Lab-Tek^®^II, catalog number 155409). Confocal fluorescence microscopy analysis was done with a TCS SP5 laser scanning confocal microscope (Leica, Vienna, Austria) equipped with a DMI6000 inverted microscope, blue diode (405 nm), Argon (458/476/488/496/514 nm), diode pumped solid state (561 nm) and HeNe (594/633 nm) lasers and PLAN APO 63× oil (NA 1.4) immersion objective lens. For flow cytometry analysis, pRBCs were diluted in PBS to a final concentration of 1−10 × 10^6^ cells/mL, and samples were analyzed using a LSRFortessa™ flow cytometer instrument (BD Biosciences, San Jose, CA, USA) set up with the five-laser, 20-parameter standard configuration, as previously described [11].

A macrophage cell line of mouse tumor origin (RAW 264.7) was grown in RPMI medium supplemented with 10% fetal calf serum, 100 µg/mL streptomycin, and 100 U/mL penicillin (complete medium) at 37 °C in an atmosphere containing 5% CO_2_. Macrophages (300 μL, 5 × 10^4^ cells/mL) were cultured in an eight-well Lab-Tek chamber slide system. After 24 h those cells that did not adhere were washed out; 48 h later, the culture was washed three times with PBS, and macrophages were treated in RPMI with 300 μL of PAA-FITC at 0.5 mg/mL. Samples were incubated for 90 min in the same conditions before washing PAAs with RPMI and staining plasma membranes with 5 µg/mL WGA-rhodamine (10 min, RT). Wells were then washed with RPMI and refilled with 300 μL complete medium before proceeding to observation of the fluorescence of FITC and rhodamine (respective λex/λem: 488/520 and 553/627 nm) with an IX51 inverted fluorescence microscope (Olympus, Tokyo, Japan). Confocal fluorescence microscopy images of [PAA-FITC]-treated macrophages were obtained as described above for RBCs, including Hoechst 33342 staining of nuclei.

### 2.5. Generation of Polyclonal Antibodies against PAAs

Immunization of rabbits was done in the facilities of the Animal Experimentation Services from the Faculty of Pharmacy at the University of Barcelona (UB) following standard protocols. The animal study was approved by the UB Animal Research Committee and the Government of Catalonia, and conducted in accordance with the Guide for the Care and Use of Laboratory Animals published by the U.S. National Institutes of Health [24]. PAAs were coupled to the carrier protein ovoalbumin and injected to two NZW rabbit females (Minimal Disease Level quality), following this immunization calendar: day 0, preimmune blood collection (reference for antibody production induced by the antigen injection) and primary immunization (intradermal injections at six to eight sites per rabbit of an aggregated amount of 250 µg PAA in a total volume of 1 mL Freund’s complete adjuvant); boosters were administered every 21 days, with the same amount of antigen emulsified in Freund’s incomplete adjuvant. Antibody titer was determined by enzyme-linked immunosorbent assay (ELISA, see below) of partial bleedings between immunizations, at days 52, 73, and 93, when the animals were euthanized and exsanguinated.

Serum antibodies to PAAs were assayed using an internal standard operating procedure. Briefly, flat-bottom 96-well ELISA plates (Nunc^®^) were coated at 4 °C overnight with 100 ng/well of antigen conjugated to bovine serum albumin (BSA) diluted in 15 mM Na_2_CO_3_, 35 mM NaHCO_3_, pH 9.6. Plates were washed with 0.1% Tween 20 in PBS (PBS-T) and then blocked with 5% skimmed milk in PBS (2 h, RT). After the plates were washed again, the test serum was diluted in 0.1% BSA, 0.05% Tween 20 in PBS, added to antigen-coated wells in triplicate, and incubated for 2 h at RT. A duplicate control dilution series of a non-hyperimmune rabbit antiserum, obtained at day zero, was also included in each plate. After extensive washing, the plates were incubated for 2 h with peroxidase-labeled goat anti-rabbit IgG (Bio-Rad, Hercules, CA, USA) diluted in the same buffer. Bound antibodies were measured by adding the substrate solution (SIGMA*FAST*™ OPD, o-phenylenediamine dihydrochloride) and determining A_450 nm_ with a Multilabel Victor3 plate reader (Perkin Elmer, Turku, Finland). A 4 Parameter Logistic (4PL) nonlinear regression model was used in all ELISA assays for the antibody titer calculation. Sera were finally stored at −20 °C until use.

Polyclonal antibodies against PAAs present in the rabbit serum (15 mg total Ig/mL) were tested in a dot blot assay. Briefly, 3 μL of 3 mg PAA/mL dilutions in ddH_2_O were applied and allowed to dry on a nitrocellulose membrane (0.45 µm, Bio-Rad, catalog number 1620145). As a positive control, 3 μL of a 1:25,000 serum dilution were spotted on the same membrane. Non-specific sites were blocked with 5% BSA in TBS-T (0.05% Tween 20, 150 mM NaCl, 20 mM Tris-HCl, pH 7.5) for 1 h at RT. Then the membrane was first incubated (30 min, RT) with rabbit plasma containing the polyclonal antibodies dissolved in TBS-T at 0.15 mg total Ig/mL (1:100 dilution), washed (TBS-T, 3× 5 min), and then treated with a secondary goat anti-rabbit antibody conjugated to horseradish peroxidase (AB6721, Abcam, Cambridge, UK) in TBS-T at 1 µg/mL (30 min, RT), followed by three 5-min washes with TBS-T and one wash with TBS (TBS-T without Tween 20). Finally, the membrane was incubated with ECL Prime Western Blotting Detection Reagent (Luminol, Amersham, UK) for 30–60 s and scanned (ImageQuant LAS4000, GE Healthcare, Chicago, IL, USA).

### 2.6. Affinity Chromatography

Half a milliliter of SulfoLink Coupling Resin, activated with iodoacetyl groups (Thermo Fisher Scientific, Waltham, MA, USA), was added to a snap cap spin column (Thermo Scientific Pierce, Waltham, MA, USA) to achieve a final column volume of 0.25 mL. For the covalent immobilization of PAAs functionalized with sulfhydryl groups (PAA-SH) on the sulfhydryl-reacting columns, these were equilibrated with coupling buffer (CB: 5 mM EDTA, 50 mM Tris-HCl, pH 8.5) and loaded with 0.5 mL of 20 mg/mL PAA-SH in CB. After gentle mixing (RT, 15 min) the columns were placed upright, and after 30 min at RT they were centrifuged (1 min, 1000 g) and washed with CB. Coupling efficacy was assessed by determining the amount of free sulfhydryl groups in the samples before and after passing through the column. Briefly, a calibration curve was prepared with dithiothreitol dissolved in 130 μL of reaction buffer (RB: 0.1 M sodium phosphate, 1 mM EDTA, pH 8.0), to which 2.5 μL of a 4 mg/mL Ellman’s reagent solution in RB were added. After 15 min incubation at RT, A_412 nm_ was determined. Then 5 mg/mL solutions of each PAA-SH in RB were processed in the same way. Unoccupied binding sites were blocked by adding 2 column volumes of quenching buffer (50 mM L-Cys-HCl in CB), followed by gentle mixing, upright incubation, centrifugation, and washing as above. Two milligrams of protein in 0.5 mL of fresh saponin extracts [25] of RBCs or pRBCs synchronized with 70% Percoll (GE Healthcare) at *Plasmodium* late stages were loaded onto each column. After mixing, upright incubation (1 h) and centrifugation as above, columns were washed with 3 to 10 column volumes of PBS. Bound proteins were eluted with a pre-warmed (55 °C) elution buffer (1× Laemmli sample buffer, supplemented with 355 mM 2-mercaptoethanol). For SDS-polyacrylamide gel electrophoresis (PAGE) analysis, samples were heated at 90 °C for 5 min in an elution buffer, and electrophoresed in 1 mm-thick 12.5% SDS-polyacrylamide gels (Mini Protean II System, Bio-Rad), which were silver-stained as previously described [26]. For the identification of PAA-binding pRBC proteins (see below), a second gel was fixed with acetic acid:ethanol:ddH_2_O (1:4:5) and stained with colloidal Coomassie Blue in 20% methanol.

### 2.7. Liquid Chromatography-Tandem Mass Spectrometry (LC-MS/MS)

Colloidal Coomassie Blue-stained gel bands were digested separately and tryptic digests were pooled to be analyzed by LC-MS/MS in a single injection. For manual in-gel digestion of proteins with trypsin, excised SDS-PAGE bands were washed sequentially with 25 mM NH_4_HCO_3_ and acetonitrile (ACN), reduced in 20 mM DTT (60 min, 56 °C), alkylated in 50 mM iodoacetamide (30 min, 30 °C, protected from light), and digested with 1.2 μg of porcine trypsin (sequencing grade modified Trypsin Gold, Promega, Madison, WI, USA) for 16 h at 37 °C. The resulting peptides were extracted from the gel matrix with 10% formic acid (FA) and ACN, and the extracts from each lane were pooled and dried in a SpeedVac concentrator.

Mass spectrometry was performed in a NanoAcquity HPLC system (Waters, Milford, MA, USA) coupled to an OrbitrapVelos mass spectrometer (Thermo Scientific). Dried extracts were taken up in 1% FA and an aliquot was injected into the liquid chromatography system equipped with a reverse-phase C18 column (75 μm internal diameter, 25 cm length, 1.7 μm particle NanoAcquity BEH column, Waters), with a mobile phase 1–40% B gradient in 60 min followed by a 40–60% B gradient in 10 min (A: 0.1% FA in water; B: 0.1% FA in ACN) and a flow rate of 250 nL/min. Eluted peptides were ionized in an emitter needle (PicoTipTM, New Objective, Zurich, Switzerland) with an applied spray voltage of 2 KV. A 300–1700 *m*/*z* range of peptide masses was analyzed in data dependent mode where a full scan was acquired with a resolution of 60,000 full width at half maximum at 400 *m*/*z*. Within this range, the 15 most abundant peptides (≥500 counts) were selected from each scan and fragmented in the linear ion trap using collision-induced dissociation (38% normalized collision energy) with He as the collision gas. The scan time settings were: Full MS: 250 ms (1 microscan) and MSn: 120 ms. Generated raw data files were collected with Thermo Xcalibur (v. 2.2).

A database was created by merging all human protein entries present in the Swiss Prot database with all entries for *Plasmodium* present in the Uniprot database (January 2016). A small database with common laboratory protein contaminants was also added and .raw data files obtained in the LC-MS/MS analyses were used to search with a SequestHT search engine using Thermo Proteome Discover (v. 1.4.1.14) against the aforementioned database. Both target and a decoy database were searched to obtain a false discovery rate (FDR), and thus estimate the number of incorrect peptide-spectrum matches that exceeded a given threshold, applying preestablished search parameters (enzyme: trypsin; missed cleavage: 5; fixed modifications: carbamidomethyl of cysteine; variable modifications: oxidation of methionine; peptide tolerance: 10 ppm and 0.6 Da for MS and MS/MS spectra, respectively). To improve the sensitivity of the database search, the semi-supervised learning machine Percolator was used in order to discriminate correct from incorrect peptide spectrum matches. Percolator assigns a q-value to each spectrum, which is defined as the minimal FDR at which the identification is deemed correct (0.01, strict; 0.05, relaxed). These q values are estimated using the distribution of scores from decoy database search.

### 2.8. Transmission Electron Microscopy (TEM) of Cell Sections

pRBC cultures were treated with 0.5 mg PAA/mL at 37 °C for 90 min, when cryosections were prepared as described [11]. Collected cryosections were incubated at RT on drops of 2% gelatin in 0.1 M PHEM buffer [27] for 30 min at 37 °C, followed by (all solutions in 0.1 M PHEM buffer) 20 mM glycine for 15 min, 10% fetal bovine serum (FBS) for 10 min, and 1% FBS for 5 min. Then they were incubated for 1 h in the presence of rabbit ISA23 antiserum diluted 1:500 in 0.1 M PHEM buffer supplemented with 1% FBS. After three washes on drops of 0.2% FBS in 0.1 M PHEM buffer for 10 min, sections were incubated for 20 min with anti-rabbit IgG coupled to 12-nm colloidal gold particles (Jackson ImmunoResearch, West Grove, PA, USA) using a 1:30 dilution in 0.1 M PHEM buffer supplemented with 1% FBS. This was followed by three washes with drops of 0.1 M PHEM buffer for 10 min, a 5-min fixation in 1% glutaraldehyde, 0.1 M PHEM buffer, and 10 1-min washes with ddH_2_O. The observations were done in a Tecnai Spirit electron microscope (FEI Company, Hillsboro, OR, USA) with a CCD SIS Megaview III camera. Controls included omission of polymer, omission of anti-PAA antibody, and staining of non-infected RBCs.

### 2.9. Determination of PAA-FITC Presence in Blood and Toxicity Assays in Mice

Inbred BALB/cAnNHsd female, 6–8-week-old mice (BALB/c; Harlan Laboratories, Indianapolis, IN, USA) were dosed orally with 100 mg/kg PAA-FITC dissolved in PBS. Then 15-μL blood samples were collected in Microvette^®^ tubes (Sarstedt) using the cross-sectional cut method, before dosing (t_0_) and at 1, 6, and 24 h post-administration. Blood samples were centrifuged for 5 min (4000 g) and stored at −20 °C; 10 μL of the supernatant were diluted 10 times with PBS and used to measure FITC fluorescence in a Spectramax Gemini XS microplate fluorimeter (Molecular Devices, LLC, Sunnyvale, CA, USA; λex/em: 485/530 nm). For in vivo toxicity assays, polymer solutions were prepared in PBS and each sample was injected intraperitoneally in three BALB/c mice; PBS was administered to control animals. Mice were individually monitored for eight days following a drug dosage regime as in the in vivo antimalarial assays performed in this work (see below). In the presence of toxic effects including, among others, >20% reduction in animal weight, aggressive and unexpected animal behavior or the presence of blood in feces, animals were immediately anesthetized using a 100 mg/kg Ketolar plus 5 mg/kg Midazolan mixture and sacrificed by cervical dislocation. Polymer maximum tolerated dose (MTD) was therefore defined upon completion of the assay as the highest dosage exhibiting an absence of the aforesaid toxicity signs. All experiments involving mice were performed in accordance with the corresponding relevant guidelines and regulations. The studies reported here were performed under protocols reviewed and approved by the Ethical Committee on Clinical Research from the *Hospital Clínic de Barcelona* (Reg. HCB/2014/0910; date of approval 14 October 2014). The animal care and use protocols followed adhered to the specific national and international guidelines specified in the Spanish Royal Decree 53/2013, which is based on the European regulation 2010/63/UE.

### 2.10. Antimalarial Activity Assay In Vivo

The in vivo antimalarial activity of free CQ and of PAA-CQ conjugates was analyzed in a four-day blood suppressive test as previously described [28]. Briefly, BALB/c mice were inoculated intraperitoneally with 2 × 10^6^ RBCs from *P. yoelii yoelii* 17XL (PyL) MRA-267-infected mice. Treatment started 2 to 4 h later (day 0) with a single dose of 5 mg CQ kg^−1^ day^−1^ administered as diphosphate-drug or PAA-drug by oral administration followed by identical dose administration for the next three days. Tested compounds were prepared in PBS and the control groups received PBS. Parasitemia was monitored daily by microscopic examination of Giemsa-stained thin blood smears. When all surviving animals had completely cleared *Plasmodium* infection they were re-infected (on day 35) with *P. yoelii* as above and left untreated. A group of animals infected and left untreated was always used as a control of a correct parasite inoculation. Survival was monitored until day 62.

### 2.11. PAA Administration to Mosquitoes

Wild *Anopheles atroparvus* mosquitoes (ca. 1000 individuals) were collected from a pig farm in the Ebre delta, in collaboration with the *Consorci de Serveis Agroambientals de les comarques del Baix Ebre i Montsià*. Females were arm-fed to produce the next generation of mosquitoes. Newly hatched female mosquitoes (F1 generation) were starved for 48 h prior to feeding them (day 0) with ISA1-FITC nanoparticles (0.125 mg/mL) incorporated in their sugar meal (0.1 mg glucose/mL), in a cage protected from light. Sugar-fed females were separated from non-fed mosquitoes and used in the experiment (continuously kept in the dark, and with access to sugar water without the labeled polymer). At different times, females were anesthetized with ether and immobilized live on a microscope slide with Vaseline and Parafilm^®^ for confocal fluorescence microscopy examination (λex/em: 495/519 nm).

Six to eight female *Anopheles gambiae* mosquitoes were tested for each PAA-FITC. The mosquitoes were removed to cups and allowed to feed for two days on 10% sucrose provided in a filter mounted on a 1-mL syringe. On day 3 the sucrose was replaced with PAA-FITC at a concentration of 0.25 mg/mL diluted in 10% sucrose. A control was included where the mosquitoes continued to feed on sucrose. After another three or five days, mosquitoes were dissected and midguts and salivary glands were fixed in 4% formaldehyde. The dissected organs were viewed under a Zeiss Axioskop 2 Plus microscope fitted with an Axiovert CCD camera (Zeiss, Oberkochen, Germany).

### 2.12. Statistical Analysis

Data are presented as the mean ± standard error of at least three independent experiments, and the corresponding standard errors in histograms are represented by error bars. Percentages of viability were obtained using non-treated cells as control of survival and IC_50_ values were calculated by nonlinear regression with an inhibitory dose-response model using GraphPad Prism5 software. Concentrations were transformed using natural log for linear regression. Regression models were adjusted for replicates and assay data.

## 3. Results

### 3.1. Characterization of PAAs and Stability Analysis in GIT Conditions

**Figure 1:**
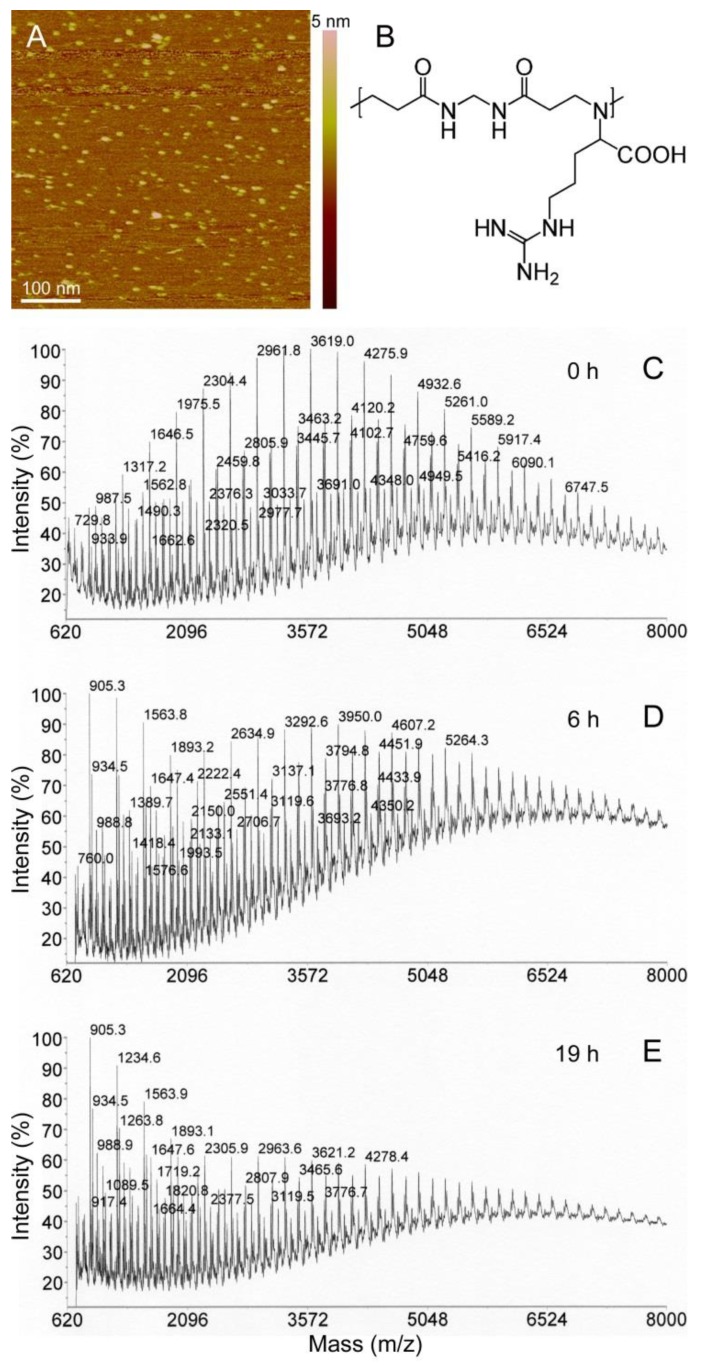
Analysis of PAA stability in GIT conditions. (**A**) AFM image in water of AGMA1. (**B**) Chemical structure of the ARGO7 repeating unit. (**C**–**E**) MALDI TOF/TOF analysis of the degradation of ARGO7 in simulated fasted-state gastric fluid.

Following synthesis, PAAs were size fractionated to have a range of molecular masses from 5 to 100 kDa, well above the maximum size previously evaluated for intraperitoneal administration (<25 kDa, [11]). AFM images taken in water on mica surfaces revealed homogeneous dispersions of particles up to 5 nm in height (Figure 1A). To evaluate their possible degradation during GIT transit, the polymers were incubated for different times in simulated fasted-state gastric fluid and subjected to MALDI TOF/TOF analysis, which permitted identification of molecules up to a molecular mass of ca. 7 kDa. Although large polymers escaped detection, this strategy allowed us to follow the degradation process over time. MALDI chromatograms exhibited regularly spaced peaks (Figure 1C), whose masses differed by discrete units that corresponded to multiples of the mass of the main building block of the respective polymers (e.g., 329 Da for ARGO7; Figure 1B). Hydrolytic degradation was evident after 6 h of incubation (Figure 1D) and reached its maximum at around 19 h (Figure 1E).

**Figure 2:**
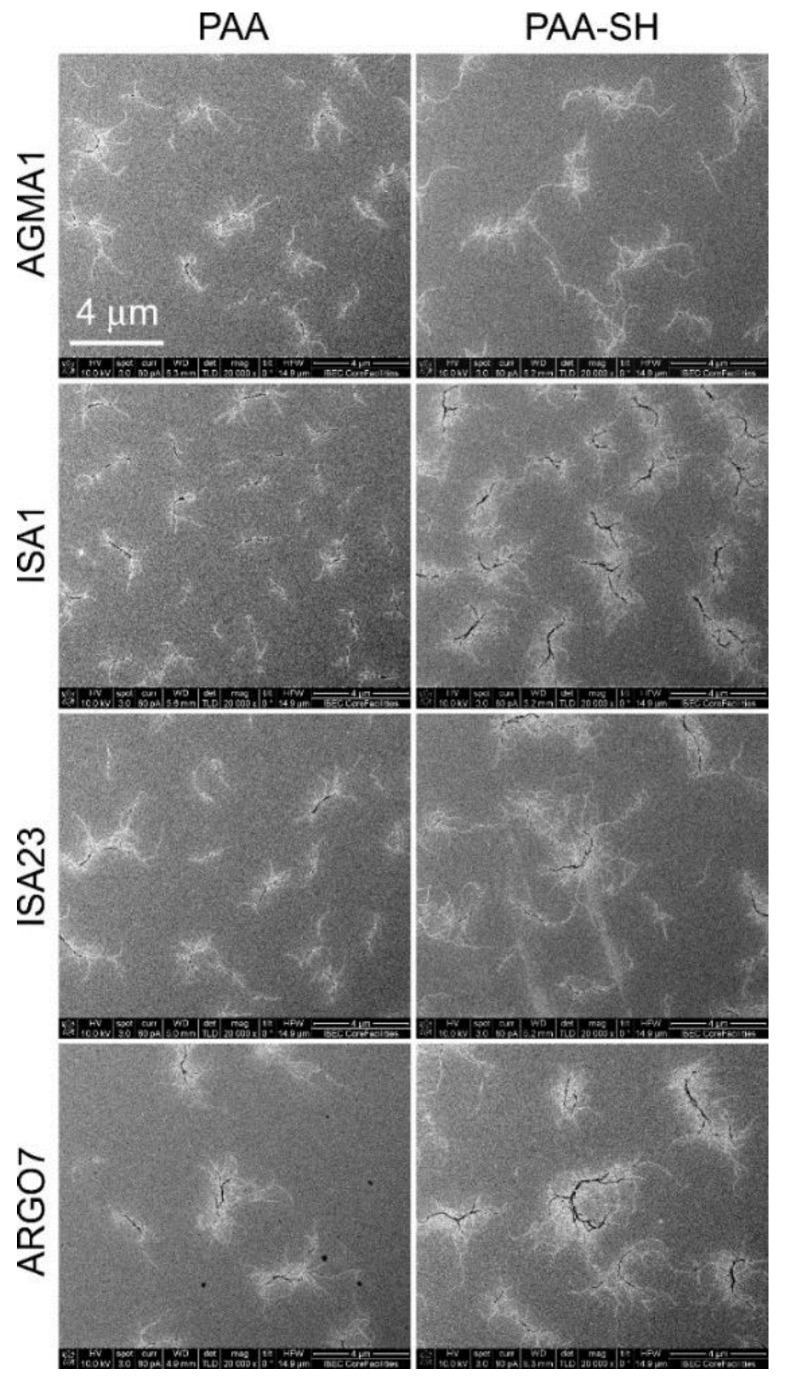
SEM analysis of PAAs, before (PAA) and after the introduction of sulfhydryl groups (PAA-SH).

The four PAAs, initially synthesized as relatively homogeneously sized preparations, acquired when dissolved in PBS a higher size and polydispersity according to nanoparticle tracking analysis (Figure S1), with estimated hydrodynamic diameters between 50 and 400 nm. In the high ionic strength of PBS, the repulsive forces between the polymer charged groups are screened, thus promoting macromolecular associations. Scanning electron microscopy analysis of the polymers dissolved in isopropanol (an aggregation-promoting solvent) revealed branched structures reaching lengths of several microns (Figure 2). PAAs have relatively large hydrodynamic volumes if compared with vinyl polymers of similar mass, indicating a tendency to assume an extended chain conformation in solution [29].

### 3.2. Cell Targeting of PAAs

As already reported for smaller polymers [11], in flow cytometry analyses PAAs did not exhibit a clear affinity for pRBCs vs. the uninfected RBCs present in the same culture (Figure S2). In complete medium little binding was observed to either cell, but in incomplete medium, PAA-cell interactions increased with both RBCs and, especially, with late-stage pRBCs (characterized by a stronger Hoechst signal due to the presence of several parasite daughter cells and therefore a larger DNA content). The differences in cell targeting observed by flow cytometry between complete and incomplete RPMI could be related to a screening effect of the proteins present in complete medium, which might prevent polymer interactions with cell membranes. Indeed, a high level of protein binding has been reported with the serum substitute Albumax used in complete RPMI [30]. This observation must be taken into account when designing in vivo experiments due to the high protein concentration found in plasma.

Confocal fluorescence microscopy examination of [PAA-FITC]-treated late-stage *P. falciparum* cultures indicated that the polymers accumulated inside the parasitophorous vacuole where the parasite is contained (Figure S3). The absence of fluorescence in the RBC cytosol of parasitized cells was consistent with the lack of FITC signal in RBCs, although according to flow cytometry data in incomplete RPMI, non-parasitized RBCs significantly interacted with some PAAs, especially with AGMA1 and ISA23 (Figure S2). Polyclonal antibodies generated against ISA23 (Figure S4) were used to investigate potential entry routes of PAAs into pRBCs. Although the antibody signal was not abundant, it could be occasionally observed associated with the plasma membrane and in intracellular areas not enclosed by a membrane (Figure S5). These observations reinforced the hypothesis that PAAs traverse the pRBC lipid bilayer through a diffusion mechanism, as previously suggested from preliminary data obtained based on the indirect detection of PAA-FITC using anti-fluorescein antibodies [11].

**Figure 3:**
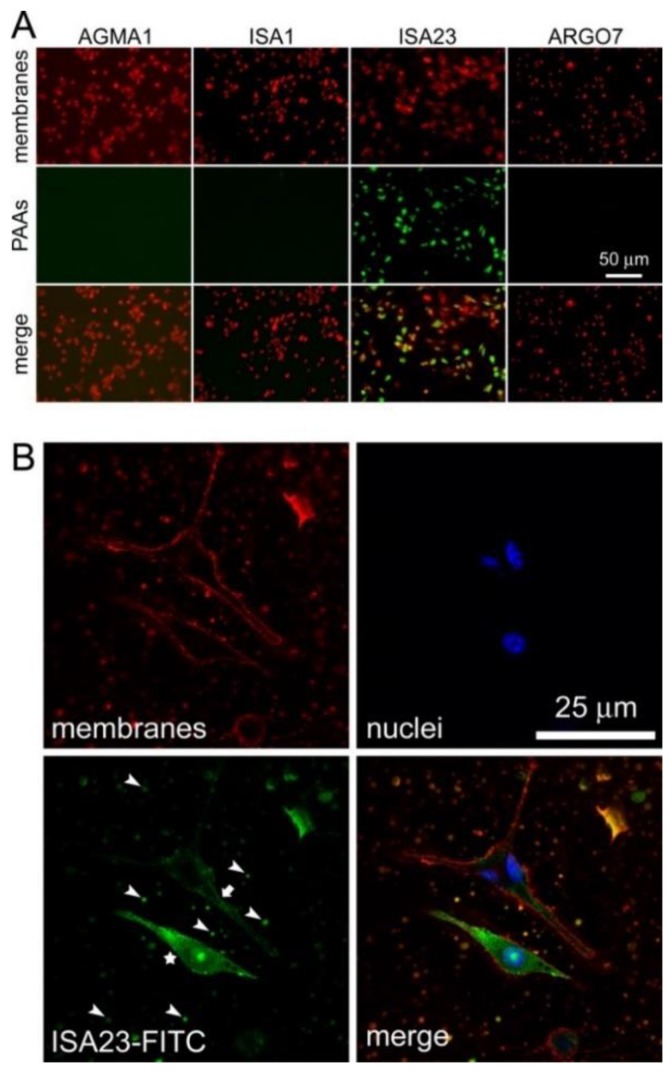
Fluorescence microscopy analysis of PAA-FITC uptake by macrophages. (**A**) Conventional fluorescence microscopy of all PAA samples. (**B**) Confocal fluorescence microscopy of the ISA23-FITC sample. Two macrophages are shown, one with intense intracellular FITC labeling (star) and another with plasma membrane labeling (arrow). Arrowheads point at some of the abundant vesicular structures present in the culture that have also incorporated the polymer.

When PAAs were incubated in the presence of macrophages in incomplete RPMI, only ISA23 was significantly taken up by the cells according to fluorescence microscopy analysis (Figure 3A). This result is consistent with the observation that ISA23 showed in the same buffer the highest interaction of all four PAAs with non-infected RBCs. Taken together, these experimental evidences indicate that ISA23 has a strong affinity for lipid bilayers, as it can be observed in confocal fluorescence microscopy images of [ISA23-FITC]-treated macrophage cultures where, in addition to cell membranes, also vesicular extracellular structures were shown to interact with the polymer (Figure 3B). Previous data showed that ISA23 had a remarkably high blood residence time [15], with about 25% of the polymer still present in the circulation 24 h after its intraperitoneal administration to mice [11]. This suggested that despite the incorporation of ISA23 into macrophages, the overall polymer concentration in blood will not be significantly affected by this cell intake.

### 3.3. Affinity Chromatography Analysis of PAA-Binding pRBC Proteins

**Figure 4:**
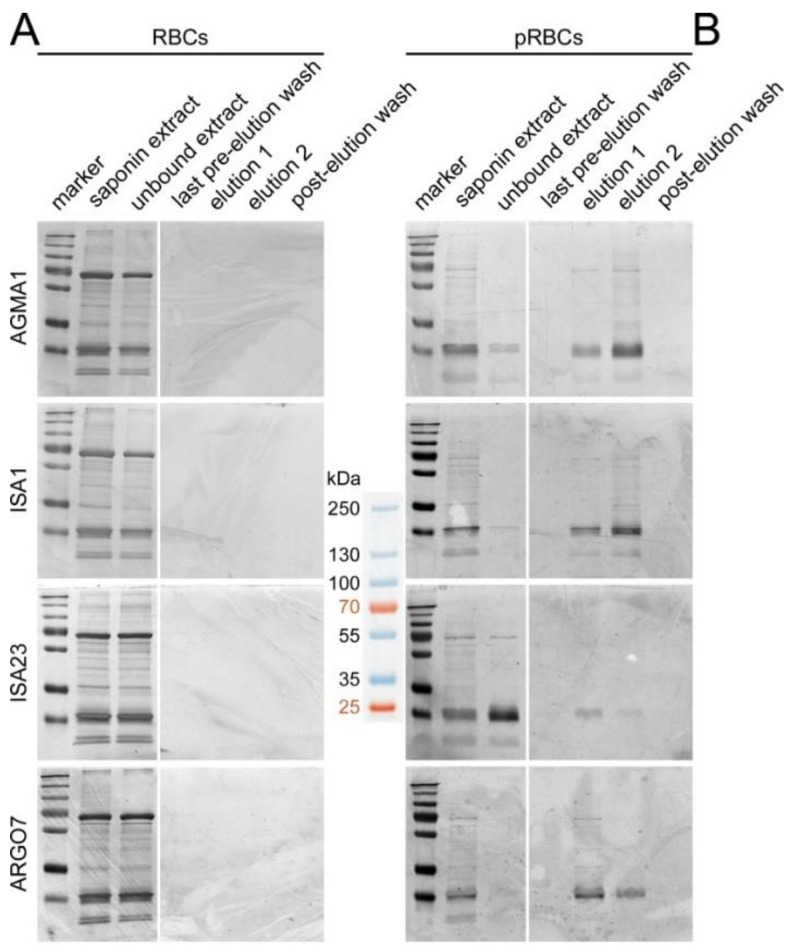
Silver-stained SDS-PAGE of pRBC saponin extracts run through four affinity chromatography columns where AGMA1, ISA1, ISA23 or ARGO7 had been immobilized. (**A**) A RBC extract was first loaded, and after the corresponding washing-elution-washing steps (**B**) a pRBC extract was loaded in the same column. The approximate masses (kDa) of the seven bands from the molecular weight marker are indicated in the space between the gels.

To investigate the molecular mechanism responsible for the preferential entry of PAAs into pRBCs vs. RBCs, an affinity chromatography approach was chosen. The four PAAs were functionalized with sulfhydryl groups that would be used to crosslink the polymers to SulfoLink columns (see Figure 2 for a SEM control of polymer structures after the incorporation of sulfhydryl groups). Coupling efficacy of PAAs to columns was found to range from 50% to 90%. According to silver-stained SDS-PAGE gels, no proteins could be detected to bind PAA-columns upon loading of uninfected RBC saponin extracts (Figure 4A). However, after loading the same columns with equal total amounts of identically prepared pRBC saponin extracts, several bands corresponding to proteins eluted from the affinity columns could be observed in the silver-stained gels of all four PAAs (Figure 4B). From a Coomassie Blue gel run in parallel with higher sample amounts loaded, the lanes from the separating gel were excised and analyzed by LC-MS/MS, where a total of 507 proteins were identified, 365 human and 142 from the *P. falciparum* 3D7 strain used. Many *Plasmodium* proteins were found to bind more than one PAA (see Table S1 for a list of *Plasmodium* proteins binding two, three, or all four PAAs), and over one-quarter (37 out of 142) interacted with the four polymers, which strongly validated the specificity of the identified PAA-protein interactions. Although the identified sequences belonged to a wide variety of families, it was remarkable the detection of several proteins involved in cell adhesion or present in the cell membrane, such as glycophorin-binding protein, erythrocyte membrane protein 3, mature parasite-infected erythrocyte surface antigen, ring-exported protein 1, surface protein P113, and high molecular weight rhoptry protein 3. We also noted the presence of several heat-shock proteins. This result strongly suggested the presence in pRBC saponin extracts of adhesive proteins interacting both with PAAs (hence the binding to affinity columns) and with RBC components (hence the presence of human RBC proteins in the fractions eluting from the columns loaded with pRBC extracts). This scenario might also explain the increased uninfected RBC targeting of some PAAs in the presence of pRBCs (Figure S2), if pRBC-secreted proteins associate with uninfected erythrocytes in the culture, e.g., through the extracellular vesicle trafficking that has been described between pRBCs [31].

**Figure 5:**
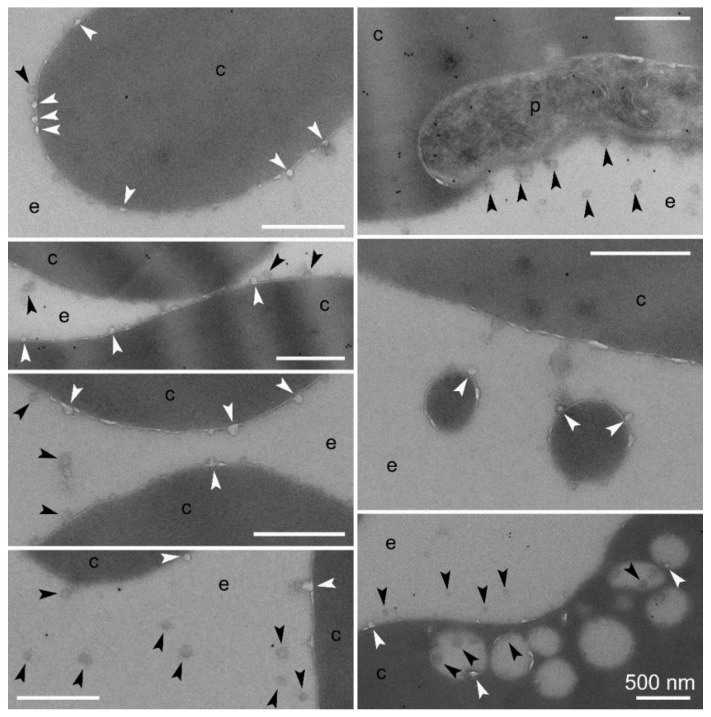
ImmunoTEM analysis of vesicular entities in *P. falciparum* in vitro cultures—e, extracellular area; c, RBC cytosol; p, *Plasmodium*. White arrowheads indicate structures in the process of budding from or merging with RBCs; black arrowheads point at vesicles not fused to RBC membranes. Size bars represent 500 nm.

In agreement with this hypothesis, we observed in immunoTEM preparations of *Plasmodium* intraerythrocytic stages numerous events of what appeared to be the budding from pRBC membranes of small vesicles below 50 nm in diameter (Figure 5). These structures, also abundantly detected in the extracellular space, seemed to be formed from protrusions of the outer bilayer leaflet of the plasma membrane, although from the static TEM images it could not be discerned whether they were leaving or entering the erythrocyte. Larger lipid bilayer-enclosed vesicles just under 500 nm in diameter that were detected in the close vicinity of the pRBC plasma membrane (both inside and outside the cell) were also observed to contain and/or be associated with the smaller vesicles. This vesicular traffic was less abundant in uninfected RBC control samples, although a detailed quantitative analysis would be required to confirm this qualitative observation.

None of the polymers was hemolytic or cytotoxic up to 1 mg/mL (Figure S6). MTD assays in mice confirmed the significant toxicity already observed for smaller ISA1 polymers at 50 mg/kg [11], whereas the other structures were well tolerated up to 200 mg/kg. This result was in agreement with previous data reporting higher toxicity for positively charged PAA dendrimers vs. their anionic counterparts after oral administration to mice [9], which led us to discard ISA1 for the preparation of in vivo tests in mice.

### 3.4. In Vitro Plasmodium Growth Inhibition Assays

**Figure 6:**
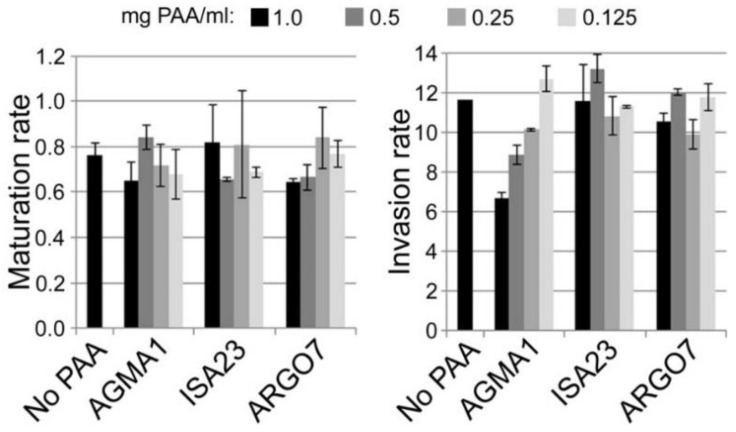
In vitro determination in the presence of PAAs of *P. falciparum* maturation and invasion rates.

CQ loaded into AGMA1, ISA23 and ARGO7 polymers reached drug payloads of 14.2%, 32.6% and 35.6% respectively; these drug contents were confirmed just before performing in vitro and in vivo assays (15.9%, 32.1% and 36.0% respectively). AFM images taken in water did not reveal significant changes in the globular structure of the polymeric nanoparticles induced by the incorporation of drug (Figure S7). SEM images after isopropanol treatment indicated that CQ loading resulted in more compact aggregated polymer structures (Figure S8), especially for those PAAs carrying more drug, which might be due to a template effect induced on the polymer by the cargo. As already described for smaller PAA structures [11], encapsulation did not improve the in vitro activity of CQ (Figure S9). Previous data reporting a modest in vitro antimalarial activity of some PAAs (particularly AGMA1) [11] led us to explore the suspected antiparasitic mechanism in more detail. Based on the known antimalarial activity of certain polymers like heparin, operating through inhibition of the RBC invasion by the parasite [32,33,34,35,36], we performed an invasion inhibition assay (Figure 6). Whereas the maturation rate of intraerythrocytic *P. falciparum* in the presence of polymers was not significantly affected up to 1 mg PAA/mL, the invasion rate in the presence of AGMA1 was clearly reduced in a dose-dependent manner, indicating that most likely the antimalarial activity mechanism of this polymer is based, as for heparin, on invasion inhibition.

### 3.5. Oral Administration to Mice

Upon oral administration to mice of PAA-FITC, fluorescence could be detected in the circulation 24 h later (Figure S10A), although we could not rule out artifacts due to potential cleavage of the PAA-FITC bond during GIT transit. However, the presence in plasma of orally administered ISA23 could be determined in dot blot assays using ISA23 antiserum (Figure S10B), indicating that at least this polymer is able to enter the blood circulation when fed to mice.

**Figure 7:**
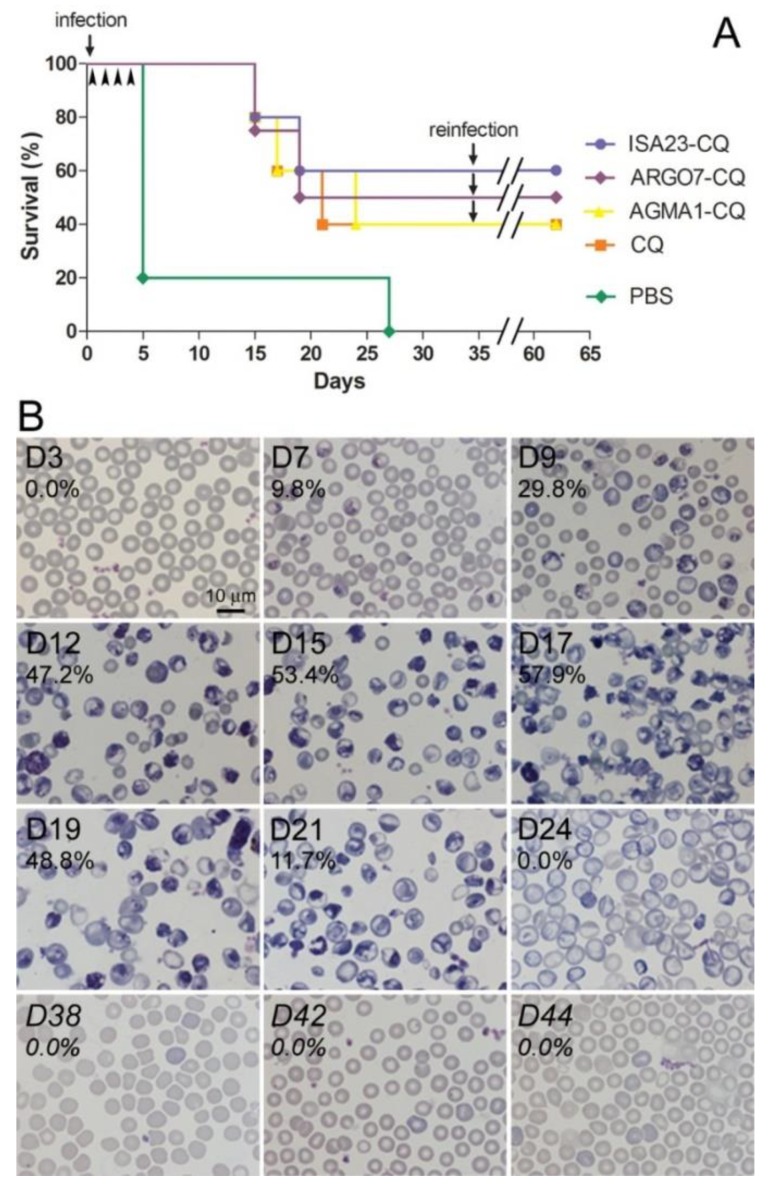
In vivo antimalarial activity assays. (**A**) Kaplan–Meier plot for the in vivo assay of the effect on *P. yoelii*-infected mice (*n* = 5 animals/sample except for ARGO7-CQ, where one of the infections was not successful) of CQ administered orally at 5 mg kg^−1^ day^−1^, either as a free drug or polymer-conjugated. (**B**) Microscopic images of blood smears prepared on different days (D0 was the day of the first infection, and reinfection was on day 35), used to determine parasitemias in a *P. yoelii*-infected mouse treated with AGMA1-CQ.

When administered orally at 5 mg CQ kg^−1^ day^−1^, PAA encapsulation only slightly improved the activity compared to the free drug (Figure 7A), with three mice cured out of five (3/5) for ISA23-CQ, two out of four for ARGO7-CQ, and two out of five for both AGMA1-CQ and free CQ. When these nine surviving animals were re-infected with *P. yoelii* and left untreated, all of them recovered completely, without presenting symptoms of the disease and with parasitemia levels below microscopy detection thresholds (Figure 7B). Control naïve infected animals left untreated developed malaria, showing high levels of parasitemia (Figure S11). This result indicated that the animals had developed immunity against *Plasmodium*, a result in agreement with previous reports of immune protection induced in malaria-infected mice that had been administered curative drug doses [23].

### 3.6. Oral Administration to Mosquitoes

**Figure 8:**
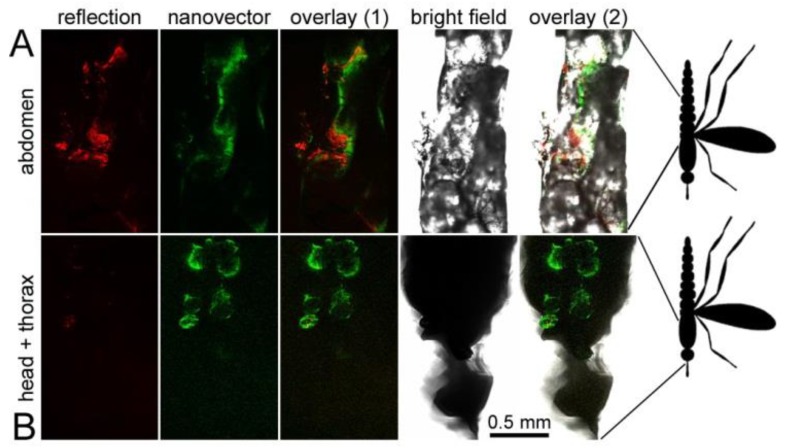
Direct delivery of PAAs to *Anopheles*. Newly hatched *A. atroparvus* were fed fluorescein-labeled ISA1 incorporated in their sugar meal. Confocal fluorescence microscopy indicated the presence of fluorescein signal (**A**) in the midgut (first day post-feeding) and (**B**) in a region of the thorax consistent with salivary gland location (three days post-feeding). In red is shown the reflection of excitation light on the cuticle of the insect.

With the aim of exploring radically new antimalarial strategies we decided to investigate if PAAs could be used in future innovative approaches against malaria involving the direct administration to mosquitoes of nanocarriers capable of encapsulating antiplasmodial drugs. To obtain additional preliminary data on the potential effect of PAAs on mosquito viability, we decided to use for this assay ISA1, which had shown the highest in vivo toxicity in mice. When *A. atroparvus* mosquitoes were fed ISA1-FITC incorporated in their sugar meal, the polymer fluorescence could be detected one day after ingestion in the insect gut (Figure 8A) and three days post-feeding in body tissues outside of the gut, including a location in the thorax, near the head, in the vicinity of the salivary glands (Figure 8B). ISA1-FITC seemed to diffuse through the midgut and circulate via the hemolymph and was detected up to six days after its ingestion, being the thorax region where fluorescence lasted longer. In a similar feeding assay done with *A. gambiae*, dissection of the mosquito organs revealed the presence of AGMA1-FITC, ARGO7-FITC and ISA23-FITC in the midgut but not in the salivary glands (Figures S12 and S13). No significant effect on mosquito life span was observed for any of the PAAs.

Preliminary targeting assays of PAA-FITC to mosquito stages of *Plasmodium* (*P. falciparum* gametocytes and *Plasmodium berghei* ookinetes, oocysts and sporozoites) did not reveal PAAs binding directly to any of these parasite forms (Figures S14–S17).

## 4. Discussion

The four PAAs assayed here interact with proteins belonging to the chaperone and glycophorin-binding families. Chaperones are required in a multitude of biological systems for the correct folding of proteins; in particular Hsp70 and Hsp90 facilitate the assembly of proteins into higher order complexes and their translocation across membranes [37]. The key role of chaperones in the cell suggests that part of the modest antimalarial activity of some PAAs might be based on their binding to intracellular components. Such a scenario is consistent with the finding that AGMA1, whose antimalarial activity is highest [11], is also the PAA associating with a larger number of *Plasmodium* proteins (Table S1). The binding of PAAs to parasite ligands is unlikely to be based on unspecific electrostatic interactions, because ISA1 and AGMA1, whose cationic character is similar (both have an average of 0.55 positive charges per unit at pH 7.4), share only about 55% of the identified affinity chromatography purified proteins. A significant portion of AGMA1 did show hepatic localization after intravenous injection in mice [15], which presents this polymer as an interesting candidate for therapies targeting the dormant liver stages (hypnozoites) of some *Plasmodium* species.

The first glycophorin-binding protein identified in *Plasmodium* was GBP130 [38], which has been shown to play a role in decreasing the membrane rigidity of pRBCs, a process involved in protein trafficking. Being glycophorin a receptor for the malaria parasite during its adhesion to red blood cells [39], the finding that PAAs associate with the parasite-encoded glycophorin-binding protein can open new therapeutic avenues based on blocking *Plasmodium*–red blood cell interactions. Invasion inhibition assays showed that AGMA1, at non-toxic concentrations that do not affect the maturation of intraerythrocytic *P. falciparum*, does reduce invasion rates in a dose-dependent manner. Among other proteins identified to bind several PAAs are the surface protein P113 and the high molecular weight rhoptry protein 2. The coupling of PAAs to these ligands might mask essential interactions of *Plasmodium* with the RBC membrane, blocking or significantly slowing down invasion. The resulting increased exposure of the parasite to the immune system might partially contribute to the protection against malaria infection observed in [PAA-CQ]-treated mice. This knowledge could be applied to the design of new antimalarial approaches where PAAs might play a dual role as carriers of drugs and as vaccination adjuvants. Regarding potential future clinical applications, it is relevant that this effect is obtained following oral intake, since this is the preferred form of administration of treatments for non-complicated malaria.

Contrary to intact erythrocytes, which lack endocytosis of large macromolecules, pRBCs develop mechanisms that confer an increased permeability to particles up to 70 nm across [40,41], which might permit the entry of PAAs below that size. This limitation leaves a narrow margin for the design of orally administered antimalarial nanocarriers, which must be synthesized with a sufficiently large size to reach the GIT without significant degradation whereas once in the blood circulation they need not be larger than a few dozen nm. PAAs, however, could be more readily adaptable to the treatment of other blood-infecting pathogens whose host cell is not the erythrocyte. The prevailingly anionic polymer ISA23 exhibited the clearest interaction with cell membranes from both RBCs and macrophages, being massively endocytosed by the latter. Such capacity presents this polymer as an interesting candidate for the coencapsulation, oral administration, and targeted delivery of drugs to treat common coinfections of pathogens parasitizing these two cell types, such as *Plasmodium* and the macrophage-infecting *Leishmania*.

The modest amelioration in the antimalarial activity of orally administered CQ provided by encapsulation in PAA polymers could be significantly improved in different ways. An increased specificity of delivery can be obtained upon functionalization of the nanocarriers with targeting molecules, e.g., antibodies against either pRBCs [20] or both parasitized and non-parasitized erythrocytes [25], or glycosaminoglycans such as heparin, which has shown specific pRBC binding [42,43]. On the other hand, coating of the nanovessels with polyethylene glycol (PEG) is a standard method used to reduce immune clearance and thereby increase circulation times allowing the carriers to reach their target site [44]. Nevertheless, this last strategy has to be more thoroughly explored because PEGylation of anionic PAA dendrimers has been described to lead to a significant decrease in intestinal cell uptake [7]. The proof-of-concept approach researched here using CQ can be extrapolated to other drugs being less efficient, more toxic, or still undiscovered. The finding that PAA encapsulation did slightly improve the exceptionally good performance of CQ as antimalarial opens perspectives for the successful application of these polymers to future orally administered therapeutic and/or prophylactic strategies.

A largely unexplored avenue in antimalarial drug development is targeting the parasite stages in the insect vector itself [45]. Preliminary data following feeding of PAA-FITC to *A. atroparvus* and *A. gambiae* indicate that the ingested polymers are not immediately excreted and have a long residence time in the midgut of both species, and in the case of *A. atroparvus* other locations in the vicinity of the salivary glands seem likely. Mosquito-dwelling parasite stages could be efficiently reached by specifically targeted nanovectors (e.g., tagged with heparin, which has been shown to bind *Plasmodium* ookinetes [43]) encapsulating the corresponding drugs. Although more studies are required, including a characterization of the longevity of nanocarriers inside the mosquito, our results suggest that encapsulated antimalarial drugs could be directly dispensed to *Anopheles* from fixed-volume containers where the drug does not become diluted with time as when it is in the blood circulation. This strategy not contemplating administration to humans would significantly reduce clinical trials and treatment development costs, thus allowing for faster transferability.

## Supplementary Materials

The following are available online at http://www.mdpi.com/1999-4923/10/4/225/s1, Figure S1: Nanoparticle tracking analysis of PAAs, Figure S2: Flow cytometry analysis of the interaction of PAAs-FITC with RBCs and pRBCs in complete and incomplete RPMI, Figure S3: Confocal fluorescence microscopy targeting study of PAAs to *P. falciparum*-containing pRBCs, Figure S4: Generation of rabbit polyclonal antibodies against ISA23, Figure S5: ImmunoTEM analysis of the subcellular localization of ISA23 in *P. falciparum* ring stages, Figure S6: PAA cytotoxicity and hemolysis assays, Figure S7: AFM images on mica of PAAs and of PAA–CQ complexes, Figure S8: SEM analysis of PAAs, Figure S9: Growth inhibition assay of the effect of PAA-encapsulated CQ on in vitro *P. falciparum* cultures, Figure S10: Detection in blood of orally administered PAAs, Figure S11: Microscopic images of blood smears prepared on days 3 and 5 post-infection from control *P. yoelii*-infected naïve mice left untreated, Figures S12 and S13: Direct delivery of PAAs to *Anopheles gambiae*, Figure S14: Targeting assay of PAA-FITC to *P. falciparum* gametocytes, Figure S15: Targeting assay of PAA-FITC to *P. berghei* ookinetes, Figure S16, Targeting assay of PAA-FITC to *P. berghei* oocysts, Figure S17: Targeting assay of PAA-FITC to *P. berghei* sporozoites, Table S1: PAA-binding proteins from the *P. falciparum* 3D7 strain identified in affinity chromatography columns.
