# Supplementary material for: Polyamidoamine Nanoparticles for the Oral Administration of Antimalarial Drugs
Source: Pharmaceutics. 2018 Nov 10;10(4):225. doi: 10.3390/pharmaceutics10040225 (PMC6321545; doi:10.3390/pharmaceutics10040225)
Supplement: Supplementary file 1 [file pharmaceutics-10-00225-s001.pdf]

pharmaceutics

MDPI

Supplementary Materials: Polyamidoamine Nanoparticles for the Oral Administration of Antimalarial Drugs

Elisabet Martí Coma-Cros, Arnau Biosca, Joana Marques, Laura Carol, Patricia Urbán, Diana Berenguer, Maria Cristina Riera, Michael Delves, Robert E. Sinden, Juan José Valle-Delgado, Lefteris Spanos, Inga Siden-Kiamos, Paula Pérez, Krijn Paaijmans, Matthias Rottmann, Amedea Manfredi, Paolo Ferruti, Elisabetta Ranucci and Xavier Fernàndez-Busquets

Supplementary Methods

1. Reagents

4-Aminobutylguanidine sulfate (97%), lithium hydroxide monohydrate (99%), 2-methylpiperazine (98%), *N,N′*-methylenebisacrylamide (96%), cystamine hydrochloride (96%), 2-mercaptoethanol (99%) and calcium chloride (97%) were purchased from Sigma-Aldrich and used as received. 2,2-bis(acrylamido)acetic acid (94%) was synthesized as previously described [1].

2. Instruments

^{1}H and ^{13}C NMR spectra were run on a Bruker Advance 400 spectrometer operating at 400.132 (^{1}H) and 100.623 (^{13}C) MHz. Size exclusion chromatography traces were obtained with a Knauer Pump 1000 equipped with a Knauer Autosampler 3800, TSKgel G4000 PW and G3000 PW TosoHaas columns connected in series, Light Scattering Viscotek 270 Dual Detector, UV detector Waters model 486, operating at 230 nm, and a refractive index detector Waters model 2410. The mobile phase was Tris-HCl buffer, pH 8.0 (0.05 M with 0.2 M sodium chloride). The flow rate was 1 mL/min and sample concentration 1% *w*/*v*.

3. Synthesis of PAAs

3.1. ISA1

Bis(acryloyl)piperazine (1.00 g, 5.15 mmol), *N,N′*-bis(hydroxyethyl)ethylenediamine (0.27 g, 2.575 mmol) and 2-methylpiperazine (0.395 g, 2.575 mmol) were dissolved in distilled water (2.0 mL) under nitrogen atmosphere and the reaction mixture was maintained at room temperature for 80 h. After this time, the product was isolated by diluting with water (3 mL), acidifying with a few drops of 12 M hydrochloric acid down to pH 4 and finally ultrafiltering through a membrane with a nominal molecular weight cutoff of 5000 Da. The retained portion was recovered by freeze-drying. Yield: 0.95 g.

1H NMR (D_2O): d = 1.08 (d, CH_3-CH); 2.46 (m, **CH**); 2.53 (m, CH_2-CON); 2.7 (m, CH_2-CH_2-CON); 3.50-3.55 (CH_2-NCO); 3.61 (CH_2-O); 3.63-3.65 (*t*-amine CH_2-N).

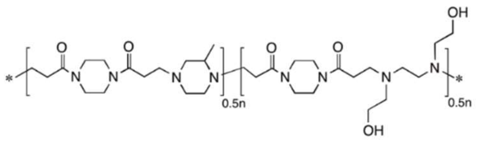

ISA1

3.2. ISA23

2,2-bis(acrylamido acetic) acid (1.00 g, 5.03 mmol) and lithium hydroxide monohydrate (0.21 g, 5.03 mmol) were dissolved in distilled water (2.0 mL) under nitrogen atmosphere, and piperazine

(0.433 g, 5.03 mmol) was then added to the obtained solution. The reaction mixture was maintained under nitrogen atmosphere at room temperature, and occasionally stirred, for 72 h. After this time, the product was isolated as described for ISA1. Yield: 1.01 g.

NMR spectral data as in [2].

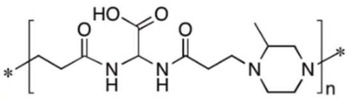

ISA23

3.3. *AGMA1*

2,2-bis(acrylamido acetic) acid (1.00 g, 5.03 mmol) and lithium hydroxide monohydrate (0.21 g, 5.03 mmol) were dissolved in distilled water (2.5 mL) under nitrogen atmosphere. Agmatine sulfate (1.18 g, 5.03 mmol) and lithium hydroxide monohydrate (0.21 g, 5.03 mmol) were then added to this solution. The reaction mixture was maintained under nitrogen atmosphere at room temperature, and occasionally stirred, for 72 h. After this time, the product was isolated as described for ISA1. Yield: 1.5 g.

NMR spectral data as in [3].

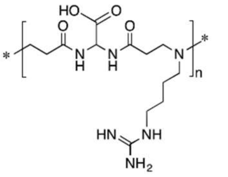

AGMA1

3.4. *ARGO7*

N,N′-methylenebisacrylamide (1.00 g, 6.22 mmol) was dispersed in water (4 mL), and L-arginine (1.095 g, 6.225 mmol) and calcium chloride were added under vigorous stirring under nitrogen atmosphere. The reaction mixture was then heated to 50 °C under stirring for 2 days. It gradually became homogeneous and was kept under nitrogen atmosphere for a further 3 weeks. The terminal double bonds in the resultant product, if any, were saturated by adding morpholine (54.48 mg, 0.622 mmol) and incubating at room temperature for a further 2 h. After this time, the product was isolated as described for ISA1. Yield: 1.00 g.

NMR spectral data as in [4].

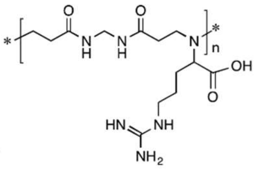

ARGO7

4. Incorporation of thiol units into PAAs

4.1. *ARGO7-SH*

L-Arginine (6.190 g, 35 mmol) and calcium chloride (0.401 g, 3.5 mmol) were added to a suspension of *N,N′*-methylenebisacrylamide (8.030 g, 50 mmol) in water (18 mL) under vigorous stirring. The reaction mixture was then heated to 50 °C until dissolution and kept under nitrogen atmosphere with gentle stirring for 1 day. After this time, the reaction mixture was cooled down to room temperature and cystamine dihydrochloride (1.759 g, 7.5 mmol) and lithium hydroxide monohydrate (0.636 g, 15 mmol) were added. The reaction mixture was kept at room temperature for 3 months and then water (40 mL) and excess mercaptoethanol (8.0 mL) were added to the hydrogel formed. Dissolution occurred after 6 days, when the solution obtained was diluted with water (100 mL), acidified to pH 4.5 with 37% hydrochloric acid and finally ultrafiltered through a membrane

with nominal molecular weight cutoff of 3000 Da. The product was recovered in 55% yield by freeze-drying the retained fraction. $\bar{M}n = 6700$, PDI = 1.48.

^{1}H NMR (D_2O): δ (ppm) = 1.58–1.95 (br, $CHC\underline{H}_2C\underline{H}_2$); 2.65-2.80 (m, $NHCOC\underline{H}_2$); 2.90-2.92 (m, $C\underline{H}_2S$); 3.17-3.30 (m, $CH_2C\underline{H}_2NH$); 3.35-3.53 (m, $NCOCH_2C\underline{H}_2N$ and $NC\underline{H}_2CH_2S$); 3.70-3.76 (m, $NC\underline{H}COOH$); 4.54-4.56 (br s,$NHC\underline{H}_2NH$).

^{13}C NMR (D_2O): δ (ppm) = 19.1 ($\underline{C}H_2S$); 24.4 ($CHCH_2\underline{C}H_2$); 25.5 ($CH\underline{C}H_2CH_2$); 30.5 ($NHCO\underline{C}H_2$); 40.8 ($CH_2\underline{C}H_2NH$); 44.3 ($NHCH_2NH$); 44.7 ($NCH_2\underline{C}H_2$); 55.2 ($N\underline{C}H_2CH_2S$); 66.6 ($\underline{C}HCOOH$); 156.8 ($H_2N\underline{C}$=NH); 172.8 (NH$\underline{C}$O); 174.1 ($\underline{C}$OOH).

AGMA1-SH and ISA23-SH were prepared as reported for ARGO7-SH, by performing the reactions at room temperature for 14 days and using the reagent amounts indicated below.

4.2. AGMA1-SH

4-aminobutylguanidine (agmatine) sulfate (3.244 g, 14.0 mmol); 2,2-bis(acrylamido)acetic acid (4.210 g, 20.0 mmol); lithium hydroxide monohydrate (1.695 g, 40.0 mmol); cystamine dihydrochloride (0.704 g, 3.0 mmol); water (18 mL). Yield 68%, $\bar{M}n = 8400$, PDI = 1.25.

^{1}H NMR (D_2O): δ (ppm) = 1.52-1.60 (br, $NCH_2CH_2C\underline{H}_2$); 1.71-1.77 (br, $NCH_2C\underline{H}_2$); 2.64-2.76 (br, $NHCOC\underline{H}_2$); 2.92-2.94 (m, $C\underline{H}_2S$); 3.08-3.20 (m, $NCOCH_2C\underline{H}_2N$); 3.34-3.47 (br, $NC\underline{H}_2CH_2CH_2$ and $NC\underline{H}_2CH_2S$); 5.51-5.56 (s, $C\underline{H}COOH$).

^{13}C NMR (D_2O): δ (ppm) = 18.5 ($\underline{C}H_2S$); 22.3 ($NCH_2CH_2\underline{C}H_2$); 25.0 ($NCH_2\underline{C}H_2$); 29.0 ($NHCO\underline{C}H_2$); 40.4 ($CH_2\underline{C}H_2NH$); 49.1 ($N\underline{C}H_2CH_2CONH$); 52.5 ($N\underline{C}H_2CH_2CH_2CH_2NH$); 54.8 ($N\underline{C}H_2CH_2S$); 56.0 ($\underline{C}HCOOH$); 155.1 ($H_2N\underline{C}$=NH); 171.3 (NH$\underline{C}$O); 173.5 ($\underline{C}$OOH).

4.3. ISA23-SH

2-methylpiperazine (1.431 g, 14.0 mmol); 2,2-bis(acrylamido)acetic acid (4.210 g, 20.0 mmol), lithium hydroxide monohydrate (1.102 g, 26.0 mmol); cystamine dihydrochloride (0.704 g, 3.0 mmol); water (7.5 mL). Yield 66%, $\bar{M}n = 9200$, PDI = 1.73.

^{1}H NMR (D_2O): δ (ppm) = 1.28 (d, CH_3); 2.55-2.65 (m, $NHCOC\underline{H}_2$); 2.80-2.90 (m, $C\underline{H}_2S$); 2.97-3.10 (m, $NCOCH_2C\underline{H}_2N$); 3.20-3.61 (br, $NC\underline{H}_2C\underline{H}_2N$, $NC\underline{H}_2C\underline{H}N$ and $NC\underline{H}_2CH_2S$); 5.48 (s, $C\underline{H}COOH$).

^{13}C NMR (D_2O): δ (ppm) = 14.0 ($\underline{C}H_3$); 20.0 ($\underline{C}H_2SH$); 30.6 ($CO\underline{C}H_2CH_2N$); 48.8 ($N\underline{C}H_2CH_2N$); 49.6 ($NCH_2\underline{C}H_2N$); 52.4 ($NCOCH_2\underline{C}H_2N$); 55.4 ($N\underline{C}H_2CHN$); 56.1 ($N\underline{C}H_2CH_2S$); 56.2 ($NCH_2\underline{C}HN$); 58.3 ($\underline{C}HCOOH$); 171.9 (NHC=O); 173.1 ($\underline{C}$OOH).

5. In vitro analysis of PAA targeting to mosquito stages of Plasmodium

5.1. Plasmodium falciparum gametocyte culture and targeting assay

Gametocytes of the *P. falciparum* NF54 strain were obtained from continuously maintained cultures of asexual blood stage parasites, setting up flasks at 1% parasitaemia and 3% hematocrit. Gametocytes began to form in significant numbers in blood culture following daily medium change. All operations were performed on a warming plate set to 37/38 °C in order to minimize heat loss to cultures during the time they were out of the incubator. Smears and an exflagellation test were performed on culture days 4, 7, 10, and 14. For the exflagellation test, a few μL of the culture were spun down and, after discarding the supernatant, the pellet was taken up in 5 μL of ookinete medium and added to a disposable counting slide; after 20 min incubation at room temperature the slide was observed under the microscope with the 40× objective. For targeting assays, 10 mL of a *P. falciparum* stage V gametocyte culture was centrifuged (37 °C, 5 min, 500 g), and the culture pellet was taken up in 3 mL of incomplete RPMI medium and added (1:1 *v/v*) to a heparin solution in the same buffer. Samples were incubated in the presence of 0.25 mg/mL PAA-FITC for 90 min at 37 °C, and the cultures were subsequently spun down and washed 3 times with incomplete RPMI medium. Smears were fixed with 4% paraformaldehyde, stained with DAPI, and examined by fluorescence confocal microscopy.

5.2. Plasmodium berghei ookinete culture and targeting assay

Ookinete culture medium consisted of 16.4 g/L RPMI supplemented with 2% *w/v* $NaHCO_3$, 0.05% *w/v* hypoxanthine, 100 μM xanthurenic acid, 50 U/mL penicillin, 50 μg/mL streptomycin (Invitrogen), 25 mM HEPES, pH 7.4. Complete medium was prepared just before use by supplementing with heat-inactivated fetal bovine serum (FBS, Invitrogen) to a final concentration of 20%. Six days prior to performing the targeting assay, a mouse was treated intraperitoneally with 10 μg/mL phenylhydrazine (PHZ) to induce reticulocytosis. Three days after PHZ treatment the mouse was inoculated by intraperitoneal injection of 200 μL of blood containing ca. 5×10^7 *P. berghei* mCherry (a kind gift from Dr. D. Vlachou) pBRCs extracted by cardiac puncture from a donor mouse that had been infected intraperitoneally 3 days before with 200 μL of a cryopreserved *P. berghei* suspension just thawed. Three days later, 1 mL of infected blood was collected by cardiac puncture onto 30 mL ookinete medium, and incubated for 24 h at 19-21 °C with 70-80% relative humidity. For ookinete targeting assays, 100 μL of 0.25 mg/mL PAA-FITC were added to 100 μL of culture and incubated in the dark for 90 min under orbital stirring (300 rpm). The samples were centrifuged for 1.5 min at 800 g and washed 3× with PBS. Fixed cell slides were prepared by adding 0.5 μL FBS to 0.5 μL pellet and by fixing the smear with 4% paraformaldehyde for 15 min. After performing 3 washing steps with PBS, slides were mounted with Vectashield® DAPI-containing media (Vector Laboratories, UK). All work involving laboratory animals was performed in accordance with the EU regulations 'EU Directive 86/609/EEC' and within the regulations of the United Kingdom Animals (Scientific Procedures) Act 1986.

5.3. P. berghei oocyst culture and targeting assay

After performing direct *Anopheles stephensi* feeding on malaria-infected mice as decribed above, for oocyst targeting assay mosquitoes were dissected for midguts on day 10 post-feeding. After anesthetizing the mosquitoes with CO_2 and keeping them on ice, they were added to ~300 μL incomplete RPMI medium and the midguts were removed under a dissecting microscope and carefully collected onto a 24-well plate containing 500 μL incomplete RPMI medium (5 midguts/well). The guts were transferred to a well containing PAA-FITC dissolved in incomplete RPMI medium at 0.25 mg/mL, and incubated for 90 min at room temperature with gentle stirring. Samples were then washed 2 times with PBS, and fixed with 4% paraformaldehyde for 15 min. After fixation, 2 washing steps with PBS were performed, and the slides were finally prepared by transferring the midguts to a slide containing a few μL of Vectashield antifade mounting medium containing DAPI.

5.4. P. berghei sporozoite culture and targeting assay

Six days prior to feeding female *A. stephensi* mosquitoes, a mouse was treated intraperitoneally with PHZ as described above, and three days after treatment the mouse was infected by intraperitoneal injection with *P. berghei* mCherry pBRCs (a kind gift from Dr. D. Vlachou). The selected female mosquitoes were not fed with sugar in the 24 h preceding the blood feed but were kept hydrated with water. On the day of the feed, the parasitemia and gametocytemia of the mouse were recorded, and an exflagellation test was also performed. Subsequently the mouse was anesthetized and placed on the netting of the mosquito cage. The feed was maintained for 30 min at 19-21 °C in the dark. After feeding, as there was a pool of sticky blood on the bottom of the cage, the pot was laid on its side overnight at 19-21 °C with 70-80% humidity. On day 1 post-feeding, the mosquitoes were anesthetized with CO_2 and kept on ice while the unfed ones were removed. Mosquitoes were then fed with 5% glucose/0.05% 4-aminobenzoic acid every 2 days and maintained for 21 days at 19-21 °C and 70-80% humidity.

For sporozoite targeting assays, mosquitoes were dissected for salivary glands on day 21 post-feeding. Glands were sliced from the head and transferred to an eppendorf tube containing 50 μL of incomplete RPMI medium. After centrifugation for 10 min at 2350 g, the pellet was taken up in 100 μL of 0.2 mg PAA-FITC/mL incomplete RPMI and incubated for 90 min at 37 °C with gentle stirring. Samples were finally centrifuged for 5 min at 2350 g and washed 3 times with incomplete RPMI. When the last wash was completed, the pellet was taken up in Hoechst 33342 solution (0.05 μg/mL)

and a 5-min centrifugation step at 2350 g was performed. The pellet was finally taken up in incomplete RPMI and the slides containing live cells were prepared for confocal microscopy analyses.

Supplementary Figures

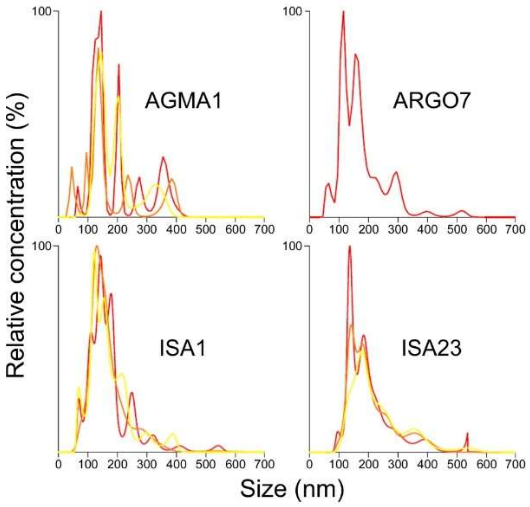

Figure S1. Nanoparticle tracking analysis of PAAs.

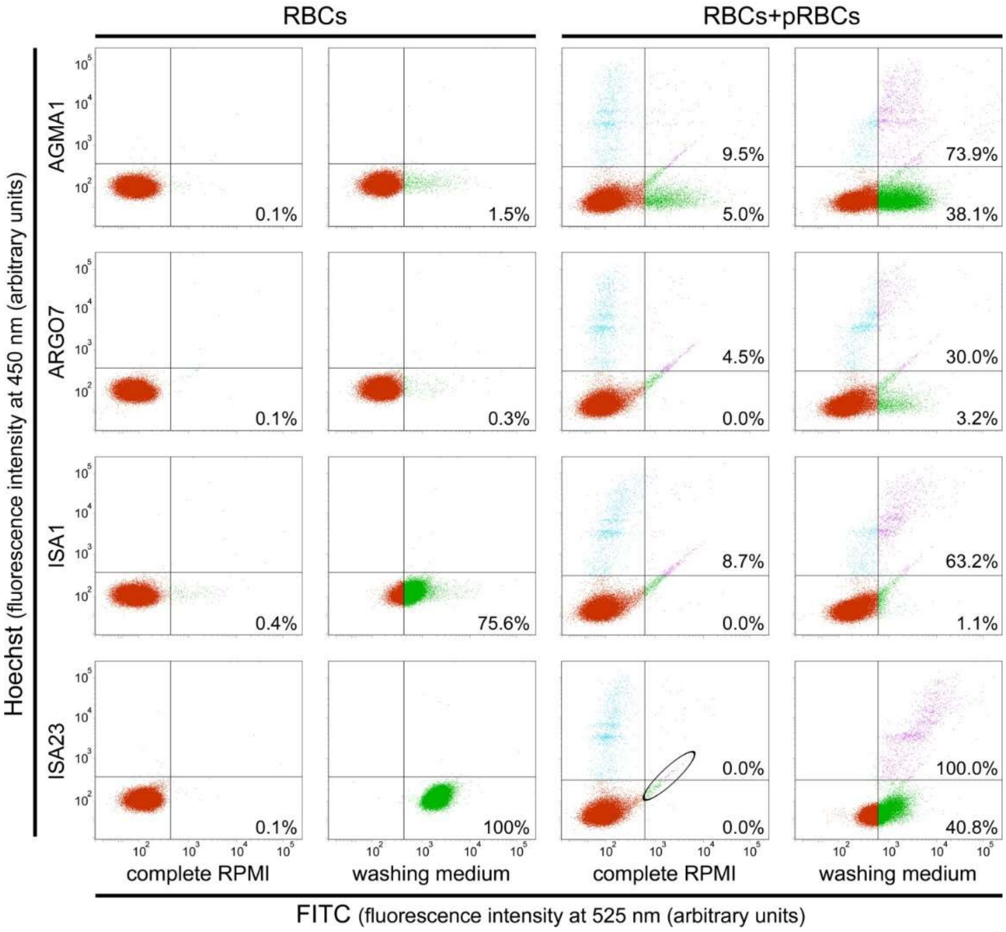

Figure S2. Flow cytometry analysis of the interaction of PAAs-FITC with RBCs and pRBCs in complete and incomplete RPMI (washing medium). The upper areas correspond to pRBCs (Hoechst fluorescence) and the right-hand areas correspond to PAA-bound cells (FITC fluorescence). Percentage values indicate the fraction of PAA-bound pRBCs (upper right areas) and RBCs (lower right areas). Unspecific fluorescence events (encircled for information only in the ISA23/RBCs+pRBCs/complete RPMI sample) were removed from the counting.

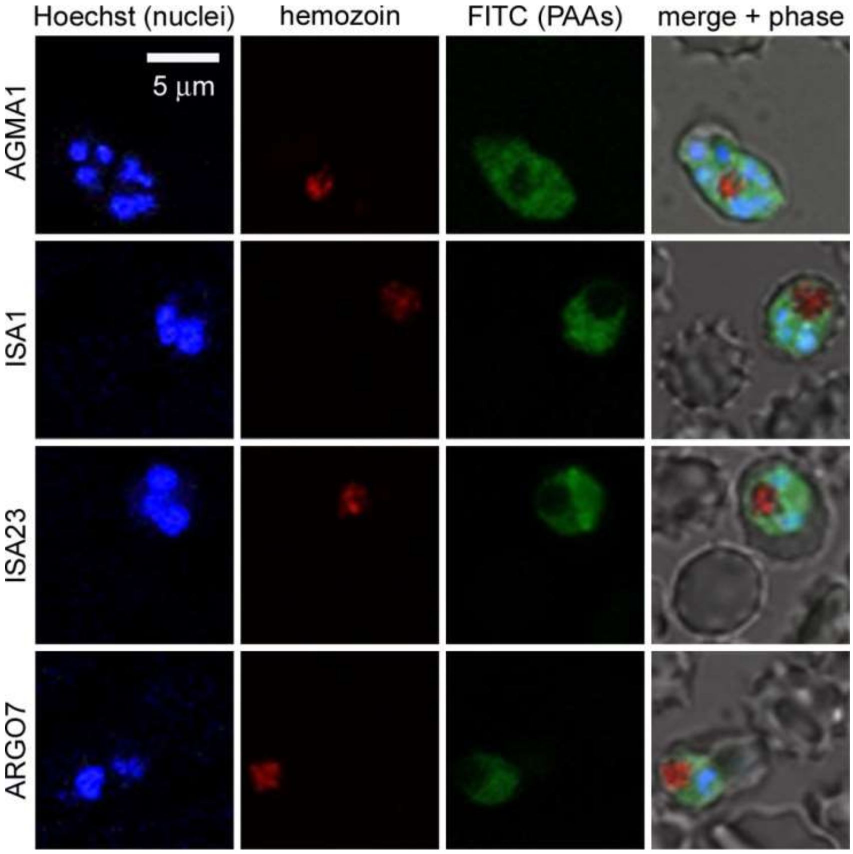

Figure S3. Confocal fluorescence microscopy targeting study of PAAs to *P. falciparum*-containing pRBCs. FITC-labeled polymers were added to living *P. falciparum* cultures of the 3D7 strain in incomplete RPMI and after 90 min of incubation the samples were processed for confocal fluorescence microscopy detection of fluorescein (PAAs), Hoechst (pRBCs), and the reflection of hemozoin crystals in pRBCs.

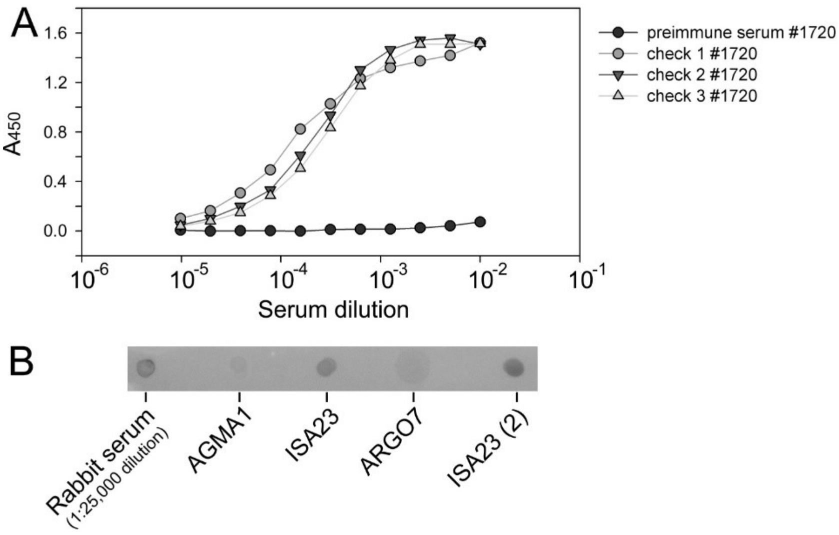

Figure S4. Generation of rabbit polyclonal antibodies against ISA23. (A) Titer determination at days 52, 73, and 93 after the initial immunization (check 1, 2 and 3, respectively). (B) Dot-blot assay to test the specificity of ISA23 antiserum. ISA23 and ISA23 (2) correspond to two different batches synthesized separately.

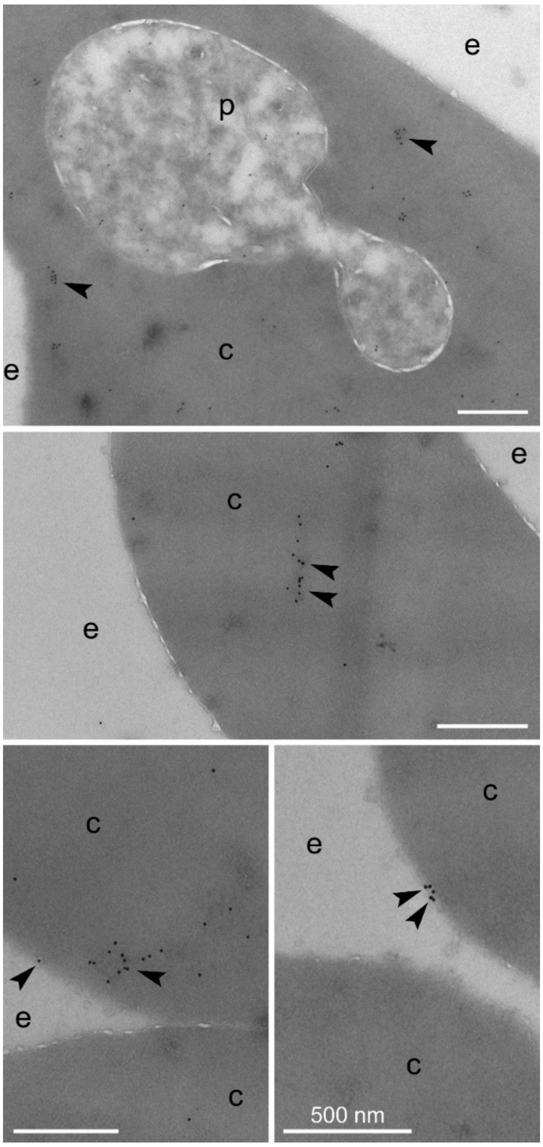

Figure S5. ImmunoTEM analysis of the subcellular localization of ISA23 in *P. falciparum* ring stages. Living pRBCs were treated overnight with ISA23, washed, and processed for immunoTEM. e, extracellular area; c, pRBC cytosol; p, *Plasmodium*. Size bars represent 500 nm.

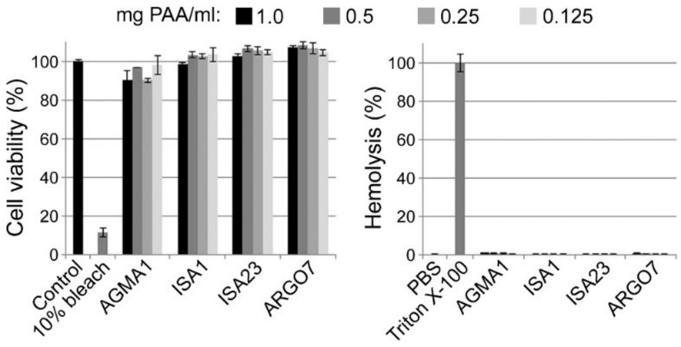

Figure S6. PAA cytotoxicity and hemolysis assays.

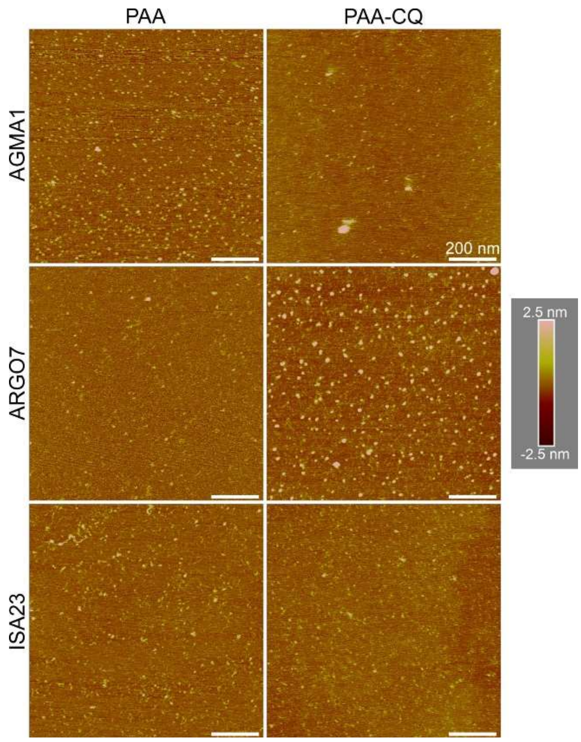

Figure S7. AFM images on mica of PAAs and of PAA-CQ complexes.

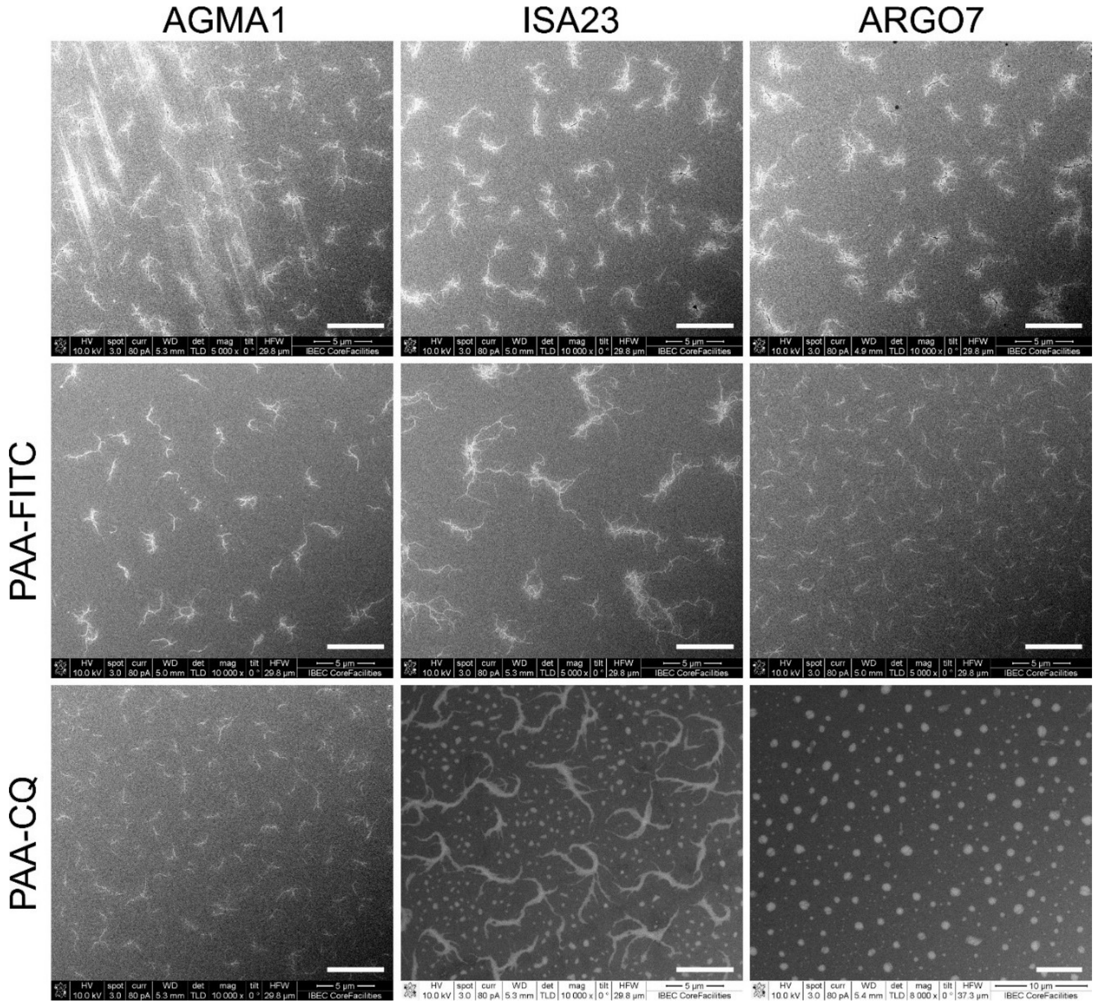

Figure S8. SEM analysis of PAAs, before (upper panels) and after the introduction of FITC groups (PAA-FITC) and the incorporation of CQ (PAA-CQ). The scale bar corresponds to 5 µm.

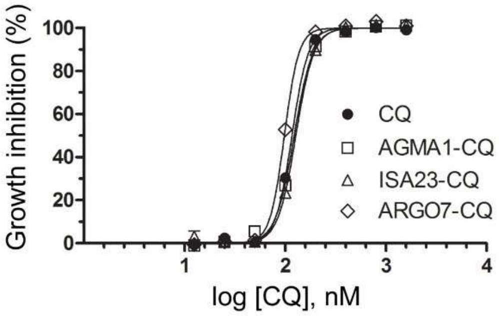

Figure S9. Growth inhibition assay of the effect on *in vitro P. falciparum* cultures of PAA-encapsulated CQ.

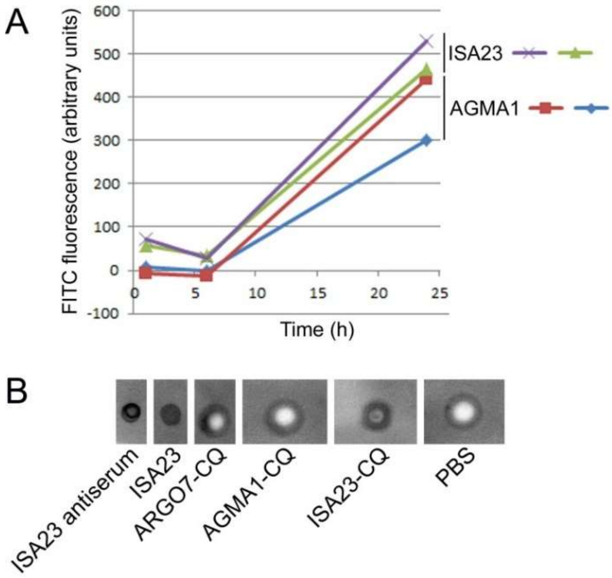

Figure S10. Detection in blood of orally administered PAAs. (A) Detection of FITC fluorescence in the plasma of mice orally-fed PAA-FITC. (B) Dot-blot assay of plasma samples obtained from mice orally-fed PAAs; for detection was used ISA23 antiserum, 3 µL of which directly spotted at a 1:25,000 dilution is shown as a control. ISA23 (3 µL of a 3 mg/mL solution) and PBS are also included as positive and negative controls, respectively.

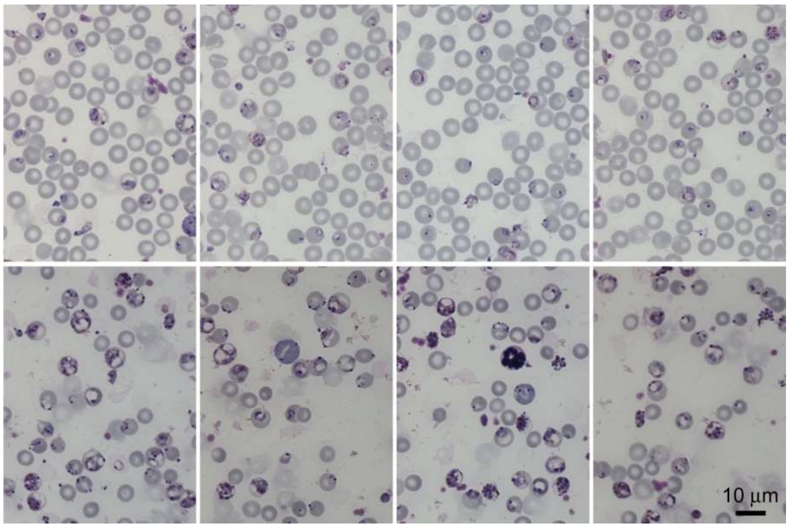

Figure S11. Microscopic images of blood smears prepared on days 3 and 5 post-infection (upper and lower row, respectively) from control *P. yoelii*-infected naïve mice left untreated.

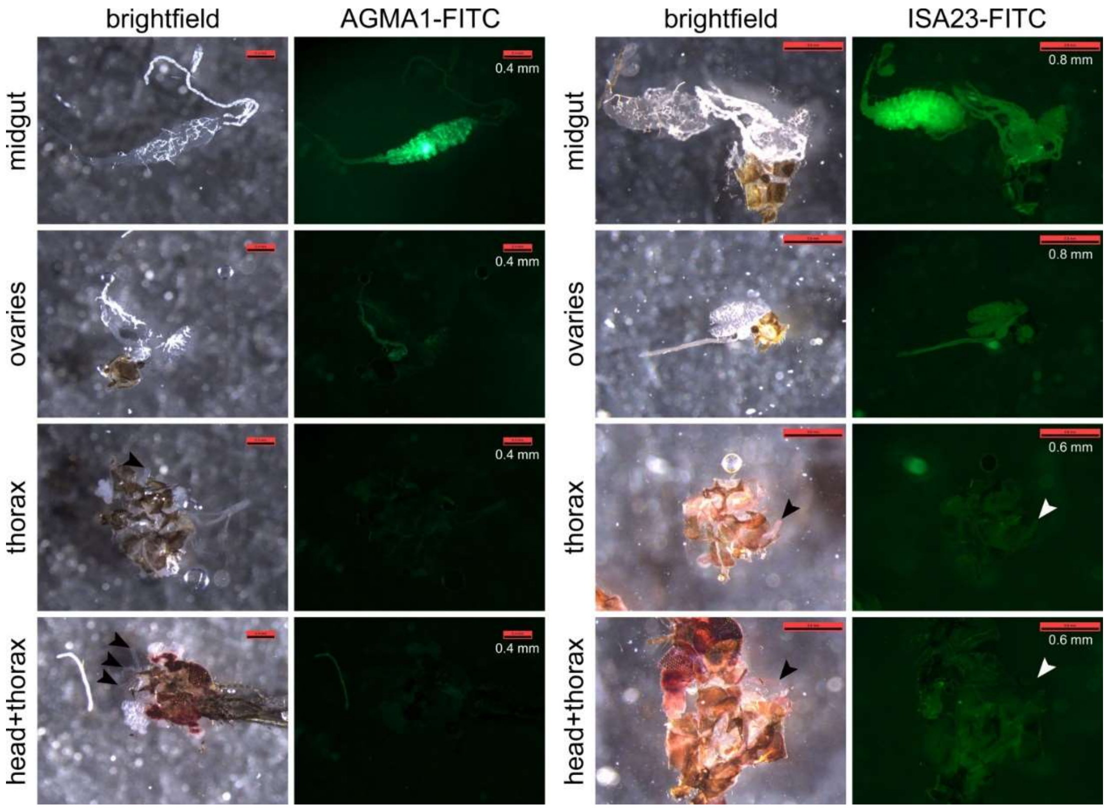

Figure S12. Direct delivery of PAAs to *Anopheles gambiae*. Fluorescein-labeled AGMA1 or ISA23 were diluted in the sugar meal provided to the mosquitoes during three or five days before proceeding to dissection and observation by fluorescence microscopy. The arrowheads indicate salivary glands.

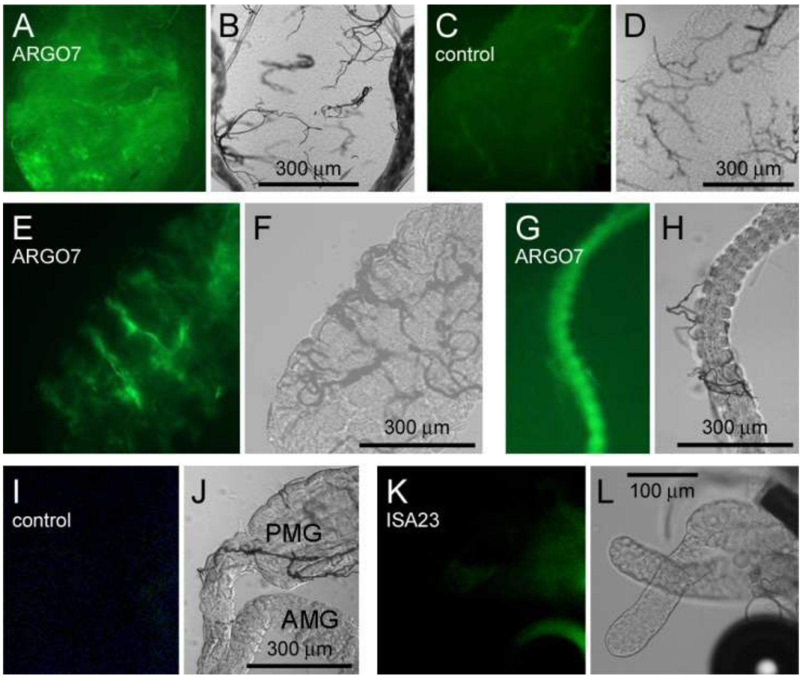

Figure S13. Direct delivery of PAAs to *Anopheles gambiae*. Fluorescein-labeled PAAs were diluted in the sugar meal provided to the mosquitoes during three or five days before proceeding to dissection and observation by fluorescence microscopy. (A) Posterior midgut from mosquito fed with ARGO7-FITC for three days. (C) Posterior midgut from control mosquitoes fed only with sugar. (E,G) Midguts (E, posterior midgut; G, anterior midgut) dissected from mosquitoes fed with ARGO7-FITC for five days. (I) Midgut from control mosquitoes fed only with sugar. Anterior midgut (AMG) and posterior midgut (PMG) are indicated. (K) Salivary glands dissected from mosquitoes fed with ISA23-FITC. Panels B, D, F, H, J and L are brightfield images of each fluorescence image immediate to the left.

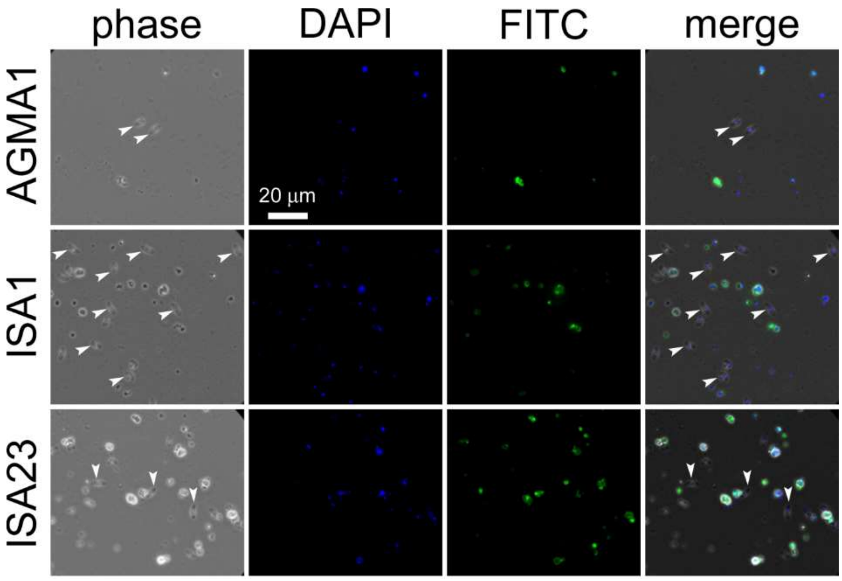

Figure S14. Targeting assay of PAA-FITC to *P. falciparum* gametocytes. Some gametocytes are indicated with arrowheads.

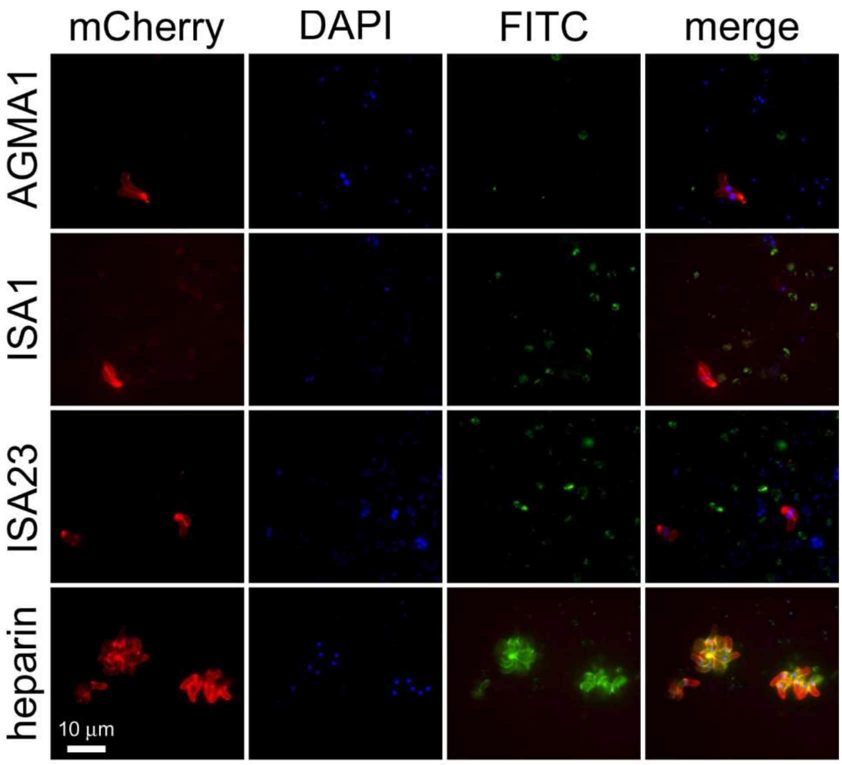

Figure S15. Targeting assay of PAA-FITC to *P. berghei* ookinetes expressing the mCherry marker. Heparin-FITC targeting is shown as a positive control [5].

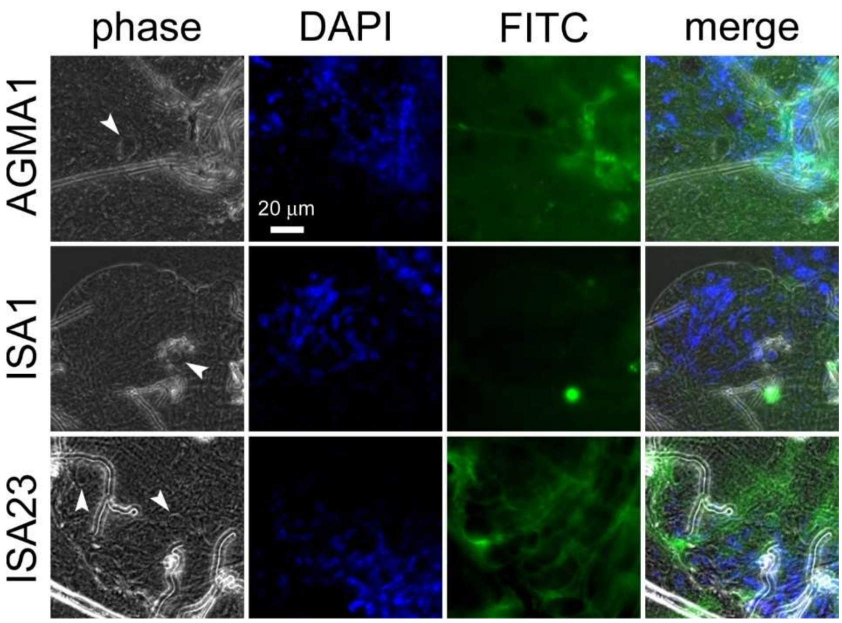

Figure S16. Targeting assay of PAA-FITC to *P. berghei* oocysts (indicated with arrowheads).

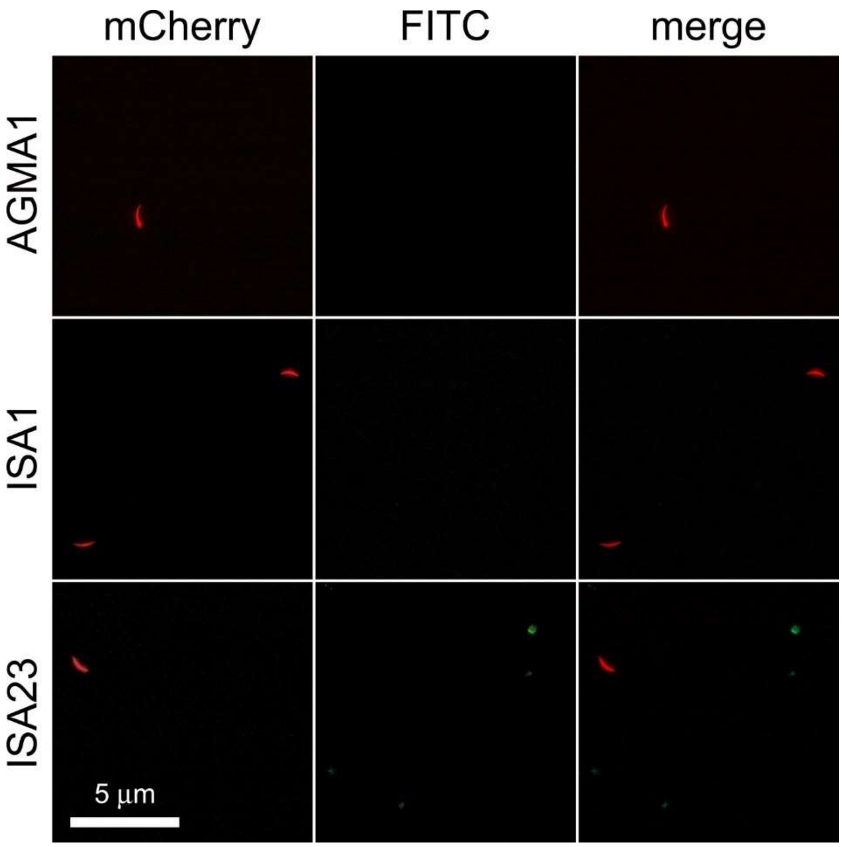

Figure S17. Targeting assay of PAA-FITC to *P. berghei* sporozoites expressing the mCherry marker.

Table S1. Proteins from the *P. falciparum* 3D7 strain identified in affinity chromatography columns and binding more than one PAA. Proteins potentially involved in extracellular interactions are highlighted in yellow and those involved in protein folding are shadowed in grey.

UniProtKB	Subcellular location	Molecular function	Biological process
**37 common proteins to all 4 PAAs**			
14-3-3 protein [C0H4V6_PLAF7]	cytoplasm / nucleus	histone binding	-
Adenosine deaminase [Q8IJA9_PLAF7]	cytosol	adenosine deaminase activity	adenosine catabolic process, hypoxanthine salvage, inosine biosynthetic process, purine ribonucleoside monophosphate biosynthetic process, response to drug
Adenosylhomocysteinase [SAHH_PLAF7]	cytosol	adenosylhomocysteinase activity / NAD binding	one-carbon metabolic process, S-adenosylhomocysteine catalytic process, S-adenosylmethionine cycle
Conserved *Plasmodium* protein [O96127_PLAF7]	-	-	-
DNA/RNA-binding protein Alba 4 [Q8IDM3_PLAF7]	cytoplasm / nucleus	DNA binding / RNA binding	-
Elongation factor 1-alpha [Q8I0P6_PLAF7]	intracellular (plasma membrane)	GTPase activity / GTP binding / translation elongation factor activity	translational elongation
Elongation factor 1-gamma, putative [Q8IDV0_PLAF7]	cytoplasm / nucleus	glutathione transferase activity / translation elongation factor	glutathione metabolic process, translational elongation
Elongation factor 2 [Q8IKW5_PLAF7]	-	GTPase activity / GTP binding / translation elongation factor activity	-
Enolase [ENO_PLAF7]	cytoplasm	magnesium ion binding / phosphopyruvate hydratase activity	activation of immune response / autophagosome assembly / glycolytic process / transcription

Eukaryotic initiation factor 4A [Q8IKF0_PLAF7]	cytoplasm / nucleus	3-5 DNA/RNA helicase activity, ATPase activity, ATP binding	regulation of gene expression, regulation of translational initiation, RNA secondary structure unwinding, translational initiation.
Fructose-bisphosphate aldolase [ALF_PLAF7]	cytoplasm	actin binding, fructose-bisphosphate aldolase activity	actin polymerization or depolymerization / glycolytic process
Glyceraldehyde-3-phosphate dehydrogenase [Q8IKK7_PLAF7]	cytosol / membrane	glyceraldehyde-3-phosphate dehydrogenase / NAD and NADP binding	gluconeogenesis / glycolytic process
Glycophorin-binding protein [GBP_PLAF7]	host cell cytoplasm	-	involved in erythrocyte invasion
Heat shock protein 110 [Q8IC01_PLAF7]	cytoplasm	ATP binding	-
Heat shock protein 70 [Q8IB24_PLAF7]	cytoplasm / nucleus	ATP binding / heat shock protein binding	-
Heat shock protein 70 [K7NTP5_PLAF7]	-	ATP binding	-
Heat shock protein 90 [Q8IC05_PLAF7]	-	ATPase activity / ATP binding / unfolded protein binding	protein folding / response to stress
Hypoxanthine phosphoribosyltransferase [Q8IJS1_PLAF7]	cytoplasm	guanine phosphoribosyltransferase activity / hypoxanthine phosphoribosyltransferase activity / metal ion binding / nucleotide binding	IMP salvage / purine ribonucleoside salvage
Inositol-3-phosphate synthase [Q8I3Y8_PLAF7]	cytoplasm	inositol-3-phosphate synthase activity	inositol biosynthetic process / phospholipid biosynthetic process

Karyopherin beta [Q8I3M5_PLAF7]	cytoplasm / nuclear membrane / nuclear periphery / nuclear pore / nuclear pore central transport channel	nuclear localization sequence binding / protein domain specific binding / protein heterodimerization activity / protein transport activity	NLS-bearing protein import into nucleus / nucleocytoplasmic transport / protein import into nucleus, translocation / ribosomal protein import into nucleus
L-lactate dehydrogenase [Q76NM3_PLAF7]	-	L-lactate dehydrogenase activity	glycolytic process
M17 leucyl aminopeptidase [Q8IL11_PLAF7]	cytosol	aminopeptidase activity / identical protein binding / manganese ion binding / metalloexopeptidase activity	-
M1-family alanyl aminopeptidase [Q8IEK1_PLAF7]	cytoplasm / food vacuole / integral component of membrane / microneme / nucleus	aminopeptidase activity / metalloaminopeptidase activity / peptide binding / zinc ion binding	peptide catabolic process / proteolysis
Ornithine aminotransferase [OAT_PLAF7]	cytoplasm	identical protein binding / ornithine-oxo-acid transaminase activity / pyridoxal phosphate binding	L-proline biosynthetic process / ornithine metabolic process
Phosphoethanolamine N-methyltransferase [Q8IDQ9_PLAF7]	Golgi apparatus	phosphoethanolamine N-methyltransferase activity	phosphatidylcholine biosynthetic process
Phosphoglycerate kinase [PGK_PLAF7]	-	ATP binding / phosphoglycerate kinase activity	glycolytic process
Phosphoglycerate mutase [Q8IIG6_PLAF7]	cytosol	2,3-bisphosphoglycerate-dependent phosphoglycerate mutase activity / phosphoglycerate mutase activity	gluconeogenesis / glycolytic process / regulation of pentose-phosphate shunt
Phosphotransferase [C6KT76_PLAF7]	cytosol	ATP binding / fructokinase activity / glucokinase activity / glucose binding / hexokinase activity / mannokinase activity	cellular glucose homeostasis / glycolytic process

Probable ATP-dependent 6-phosphofructokinase [Q8I2Z8_PLAF7]	cytoplasm	6-phosphofructokinase activity / ATP binding / diphosphate-fructose-6-phosphate 1-phosphotransferase activity / metal ion binding	fructose 6-phosphate metabolic process
Proteasome subunit alpha type [Q8IBI3_PLAF7]	cytoplasm / nucleus	endopeptidase activity / threonine-type endopeptidase activity	proteasome-mediated ubiquitin-dependent protein catabolic process / ubiquitin-dependent protein catabolic process
Pyruvate kinase [C6KTA4_PLAF7]	cytoplasm	kinase activity / magnesium ion binding / potassium ion binding / pyruvate kinase activity	protein homotetramerization
Receptor for activated kinase C [Q8IBA0_PLAF7]	cytosol	kinase activity / protein kinase C binding	-
S-adenosylmethionine synthase [Q7K6A4_PLAF7]	cytosol	ATP binding / metal ion binding / methionine adenosyltransferase activity / protein homodimerization activity	one-carbon metabolic process / S-adenosylmethionine biosynthetic process
Serine-repeat antigen protein [SERA_PLAF7]	parasitophorous vacuole (extracellular space / lysosomes / symbiont-containing vacuole)	cysteine-type endopeptidase activity / cysteine-type peptidase activity	immunoglobulin production / proteolysis / proteolysis involved in cellular protein catabolic process / regulation of immune response
STI1-like protein [STI1L_PLAF7]	cytosol	-	-
T-complex protein 1 subunit alpha [Q8II43_PLAF7]	cytoplasm	ATP binding / protein binding involved in protein folding / unfolded protein binding	de novo protein folding / chaperone-mediated protein folding / protein folding

Thioredoxin peroxidase 1 [Q8IL80_PLAF7]	cytosol	antioxidant activity / protein dimerization activity / thioredoxin peroxidase activity	cell redox / DNA protection / response to heat / response to oxidative stress
**30 common proteins to AGMA1, ISA1 and ARGO7**			
Actin-1 [ACT1_PLAF7]	cytoskeleton	ATP binding / structural constituent of cytoskeleton	actin polymerization-dependent cell motility involved in migration of symbiont in host / cytoskeleton organization
ATP-dependent RNA helicase [Q9TY94_PLAF7]	cytoplasm / nucleolus / nucleus / spliceosomal complex	ATP binding / ATP-dependent RNA helicase activity / RNA binding	cellular response to DNA damage stimulus / mRNA export from nucleus / mRNA splicing, via spliceosomes / regulation gene expression / RNA secondary structure unwinding
Cell division cycle protein 48 homologue, putative [C6KT34_PLAF7]	-	ATP binding / hydrolase activity	cell division
EMP1-trafficking protein [Q8IBF2_PLAF7]	transmembrane (integral component of membrane)	-	-
Erythrocyte membrane protein 3 [O96124_PLAF7]	transmembrane (cytoplasm integral component of membrane/ Maurer's cleft/ symbiont-containing vacuole)	-	-
Falcilysin [Q76NL8_PLAF7]	apicoplast / food vacuole	metal ion binding / metalloendopeptidase activity	hemoglobin catabolic process
Glutathione reductase [GSHR_PLAF7]	cytoplasm (apicoplast/ cytoplasm)	electron transfer activity / flavin adenine dinucleotide binding / glutathione-disulfide reductase activity / thioredoxin-disulfide reductase activity	cell redox homeostasis / response to oxidative stress / response to oxygen radical

Glycerophosphodiester phosphodiesterase, putative [Q8IM31_PLAF7]	cytosol / food vacuole / symbiont-containing vacuole	glycerophosphodiester phosphodiesterase activity	lipid metabolic process
GTP-binding nuclear protein [Q7KQK6_PLAF7]	nucleus	GTPase activity / GTP binding	intracellular protein transport / protein import into nucleus / regulation of cell cycle / ribosomal subunit export from nucleus / RNA export from nucleus
Haloacid dehalogenase-like hydrolase [Q8IJ74_PLAF7]	cytoplasm	metal ion binding / sugar-phosphatase activity	dephosphorylation / negative regulation of isopentenyl diphosphate biosynthetic process, methylerythritol 4-phosphate pathway
Haloacid dehalogenase-like hydrolase, putative [Q8I5F4_PLAF7]	-	hydrolase activity	-
Heat shock protein 70 [Q8I2X4_PLAF7]	endoplasmic reticulum	ATP binding	-
Mature parasite-infected erythrocyte surface antigen [Q8I492_PLAF7]	host cell plasma membrane	-	-
Obg-like ATPase 1 [Q8IBM9_PLAF7]	cytoplasm	ATPase activity / ATP binding / GTP binding / ribosomal large subunit binding / ribosome binding	signal transduction
Peptidase, putative [Q8IKT5_PLAF7]	cytosol / food vacuole	metal ion binding / metalloaminopeptidase activity / protein homodimerization activity	hemoglobin catabolic process / proteolysis
Probable inorganic pyrophosphatase [IPYR_PLAF7]	cytoplasm	inorganic diphosphatase activity / magnesium ion binding	phosphate-containing compound metabolic process

Protein DJ-1 [C6KTB1_PLAF7]	cytosol	hydroxyethylthiazole kinase activity / peptidase activity	protein folding / thiamine biosynthetic process
Pyridoxal 5'-phosphate synthase subunit Pdx1 [PDX1_PLAF7]	cytoplasm / cytosol	catalytic activity / glutaminase activity / identical protein binding / pyridoxal 5'-phosphate synthase	pyridoxal phosphate biosynthetic process / pyridoxine biosynthetic process / response to singlet oxygen / vitamin B6 biosynthetic process
Ring-exported protein 1 [Q8I2G1_PLAF7]	transmembrane (cytoplasm/ host cell membrane/ integral component of membrane/ Maurer's cleft)	-	-
S-antigen protein [SANT_PLAF7]	parasitophorous vacuole (symbiont-containing vacuole)	-	-
Serine repeat antigen 3 [O96165_PLAF7]	extracellular space / lysosome	cysteine-type endopeptidase activity / cysteine-type peptidase activity	immunoglobulin production / proteolysis / proteolysis involved in cellular protein catabolic process / regulation of immune response
Serine repeat antigen 4 [O96164_PLAF7]	extracellular space / lysosome / symbiont-containing vacuole	cysteine-type endopeptidase activity / cysteine-type peptidase activity	immunoglobulin production / proteolysis / proteolysis involved in cellular protein catabolic process / regulation of immune response
Serine repeat antigen 7 [O96163_PLAF7]	extracellular space / lysosome	cysteine-type endopeptidase activity / cysteine-type peptidase activity	immunoglobulin production / proteolysis / proteolysis involved in cellular protein catabolic process / regulation of immune response
Serine repeat antigen 9 [Q8I3C0_PLAF7]	extracellular space / lysosome	cysteine-type endopeptidase activity	immunoglobulin production / proteolysis involved in cellular protein catabolic process / regulation of immune response

Surface protein P113 [Q8ILP3_PLAF7]	transmembrane (integral component of membrane/ symbiont-containing vacuole membrane)	-	-
T-complex protein 1 subunit beta [O97247_PLAF7]	cytoplasm	protein binding involved in protein folding / unfolded protein binding	-
T-complex protein 1 subunit delta [C0H5I7_PLAF7]	cytoplasm	ATP biding / protein binding involved in protein folding / unfolded protein binding	de novo protein folding / chaperone-mediated protein folding / protein folding
T-complex protein 1 subunit eta [TCPH_PLAF7]	cytoplasm	ATP biding / protein binding involved in protein folding / unfolded protein binding	de novo protein folding / chaperone-mediated protein folding / protein folding
T-complex protein 1 subunit gamma [Q8I5C4_PLAF7]	cytoplasm	ATP biding / protein binding involved in protein folding / unfolded protein binding	de novo protein folding / chaperone-mediated protein folding / protein folding
Tubulin beta chain [TBB_PLAF7]	cytoskeleton (microtube)	GTPase activity / GTP binding / structural constituent of cytoskeleton	microtubule cytoskeleton organization / response to drug
**2 common proteins to AGMA1, ISA23 and ARGO7**			
High molecular weight rhoptry protein 2 [C0H571_PLAF7]	rhoptry	-	-
Polyadenylate-binding protein [Q8I5H4_PLAF7]	cytoplasm	mRNA binding / poly (A) binding	RNA processing
**2 common proteins to AGMA1, ISA1 and ISA23**			
Methionine-tRNA ligase [Q8IJ60_PLAF7]	cytoplasm	ATP binding / methionine tRNA ligase activity	methionyl-tRNA aminoacylation
T-complex protein 1 subunit epsilon [O97282_PLAF7]	cytoplasm	ATP binding / protein binding involved in protein folding / unfolded protein binding	de novo protein folding / chaperone-mediated protein folding / protein folding

**1 common protein to ISA1, ISA23 and ARGO7**			
Triosephosphate isomerase [TPIS_PLAF7]	cytosol	triose-phosphate isomerase activity	fatty acid biosynthetic process / gluconeogenesis / glyceraldehyde-3-phosphate biosynthetic process / glycerol catabolic process / glycolytic process / pentose-phosphate shunt
**6 common proteins to AGMA1 and ISA1**			
Asparagine-tRNA ligase [O96198_PLAF7]	cytoplasm	asparagine-tRNA ligase activity / ATP binding / nucleic acid binding	asparaginyl-tRNA aminoacylation
Ethanolamine kinase [Q8IIB7_PLAF7]	cytoplasm	ethanolamine kinase activity	lipid metabolic process / phosphatidylcholine biosynthetic process / phosphatidylethanolamine biosynthetic process
Proliferation-associated protein 2g4, putative [Q8ILI2_PLAF7]	-	metalloexopeptidase activity	cell cycle arrest / cell proliferation
Serine hydroxymethyltransferase [Q8I566_PLAF7]	apicoplast / cytoplasm / mitochondrion	glycine hydroxymethyltransferase activity / methyltransferase activity / pyridoxal phosphate binding	D-serine metabolic process / glycine biosynthetic process from serine / one-carbon metabolic process / tetrahydrofolate interconversion
T-complex protein 1 subunit theta [O96220_PLAF7]	cytoplasm	1-phosphatidylinositol-3-phosphate 5-kinase activity / ATP binding	phosphatidylinositol phosphorylation / protein folding / retrograde transport (endosome to Golgi)
Ubiquitin-activating enzyme E1 [Q8I5F9_PLAF7]	cytoplasm	ubiquitin activating enzyme activity	cellular response to DNA damage stimulus / ubiquitin-dependent protein catabolic process

**4 common proteins to AGMA1 and ISA23**			
Cytoadherence linked asexual protein 3.2 [O77309_PLAF7]	host cell plasma membrane / rhoptry	cell adhesion molecule binding	cytoadherence to microvasculature (mediated by symbiont protein)
Eukaryotic translation initiation factor 3 subunit M [Q8I1Q5_PLAF7]	cytoplasm (eukaryotic 43S preinitiation complex)	translation initiation factor activity	cytoplasmic translation initiation / formation of translation preinitiation complex / regulation of translational initiation
Importin subunit alpha [Q8IAW0_PLAF7]	cytosol / nuclear pore / nuclear pore central transport channel / nucleoplasm	nuclear import signal receptor activity / nuclear localization sequence binding / protein domain specific binding / protein heterodimerization activity / protein transport activity	NLS-bearing protein import into nucleus / nuceocytoplasmic transport
Rab GDP dissociation inhibitor [Q8I501_PLAF7]	intracellular	GTPase activator activity / Rab GDP-dissociation inhibitor activity	protein transport / small GTPase mediated signal transduction / vesicle-mediated transport
**20 common proteins to AGMA1 and ARGO7**			
101 kDa malaria antigen [ABRA_PLAF7]	merozoite cell surface / parasitophorous vacuole	-	-
60S acidic ribosomal protein P0 [Q8II61_PLAF7]	cytosolic large ribosomal subunits	structural constituent ribosome	ribosome biogenesis / translation
Acyl-CoA synthetase [Q8I6Z1_PLAF7]	-	AMP binding / decanoate-CoA ligase activity / long-chain fatty acid-CoA ligase activity	fatty acid metabolic process
ADP-ribosylation factor 1 [ARF1_PLAF7]	Golgi apparatus	GTP binding	protein ADP-ribosylation / protein transport / vesicle-mediated transport
Alpha/beta hydrolase, putative [Q8IK20_PLAF7]	membrane	acylglycerol lipase activity / lipase activity	lipid metabolic process

Duffy binding-like merozoite surface protein [Q8IJ52_PLAF7]	cell surface / integral component of membrane	host cell surface binding / receptor activity	entry into host cell / pathogenesis
Glutamate-tRNA ligase, putative [Q8IDK7_PLAF7]	cytoplasm	ATP binding / glutamate-tRNA ligase activity	glutamyl-tRNA aminoacylation
GTPase-activating protein, putative [Q8I3A9_PLAF7]	-	-	-
High molecular weight rhoptry protein 3 [Q8I395_PLAF7]	rhoptry	-	entry into host cell
Merozoite surface protein P41 [PF41_PLAF7]	merozoite cell surface / cell membrane	miscellaneous	miscellaneous
Nucleosome assembly protein [Q8I608_PLAF7]	cytoplasm / nucleus	histone binding	nucleosome assembly
Parasitophorous vacuolar protein 1 [Q8II72_PLAF7]	symbiont-containing vacuole	-	-
PRE-binding protein [Q8IJS7_PLAF7]	nucleus	DNA binding transcription factor activity / double-stranded DNA binding / RNA binding	regulation of transcription (DNA-templated)
Proliferating cell nuclear antigen [PCNA_PLAF7]	nucleus	DNA binding / DNA polymerase processivity factor activity	leading strand elongation / mismatch repair / regulation of DNA replication / translesion synthesis
Proteasome endopeptidase complex [C6KST3_PLAF7]	nucleus / cytoplasm / proteasome core complex	endopeptidase activity / threonine-type endopeptidase activity	ubiquitin-dependent protein catabolic process
Ribonucleotide reductase small subunit [Q8IM38_PLAF7]	-	ribonuleoside-diphosphate reductase activity	deoxyribonucleotide biosynthetic process
Serine repeat antigen 6 [Q9TY96_PLAF7]	extracellular space / lysosome / symbiont-containing vacuole	cysteine-type endopeptidase activity / cysteine-type peptidase activity	immunoglobulin production / proteolysis

T-complex protein 1 subunit zeta [C6KST5_PLAF7]	cytoplasm	ATP binding / protein binding involved in protein folding / unfolded protein binding	de novo protein folding / chaperone-mediated protein folding / protein folding
Tubulin alpha chain [Q6ZLZ9_PLAF7]	cytoskeleton	GTPase activity / GTP binding / structural constituent of cytoskeleton	microtubule-based process
V-type proton ATPase subunit B [Q6ZMA8_PLAF7]	cell / proton-transporting V-type ATPase (V1 domain)	ATP binding / proton-transporting ATPase activity	ATP hydrolysis coupled proton transport / ATP metabolic process / vacuolar acidification
**1 common protein to ISA1 and ARGO7**			
1-Cys peroxiredoxin [Q8IAM2_PLAF7]	cytosol	antioxidant activity / peroxidase activity / peroxiredoxin activity	cell redox homeostasis / response to oxidative stress
**1 common protein to ISA23 and ARGO7**			
Purine nucleoside phosphorylase [Q8I3X4_PLAF7]	-	purine-nucleoside phosphorylase activity	nucleoside metabolic process

Venn diagram showing the distribution of the 142 *Plasmodium* proteins binding the four PAA columns:

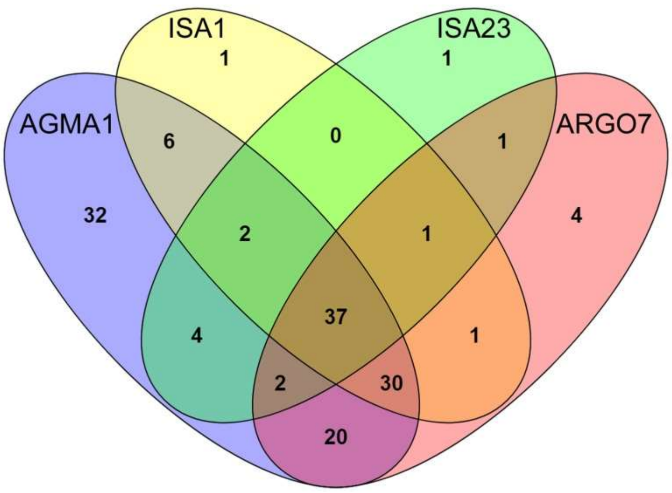

References

1. Ferruti, P.; Ranucci, E.; Trotta, F.; Gianasi, E.; Evagorou, E.G.; Wasil, M.; Wilson, G.; Duncan, R. Synthesis, characterisation and antitumour activity of platinum(II) complexes of novel functionalised poly(amido amine)s. *Macromol. Chem. Phys.* **1999**, *200* (7), 1644-1654, https://doi.org/10.1002/(SICI)1521-3935(19990701)200:7%3C1644::AID-MACP1644%3E3.0.CO;2-P.
2. Ranucci, E.; Ferruti, P.; Lattanzio, E.; Manfredi, A.; Rossi, M.; Mussini, P.R.; Chiellini, F.; Bartoli, C. Acid-base properties of poly(amidoamine)s. *J. Polym. Sci. A Polym. Chem.* **2009**, *47* (24), 6977-6991, http://dx.doi.org/10.1002/pola.23737.
3. Ferruti, P.; Franchini, J.; Bencini, M.; Ranucci, E.; Zara, G.P.; Serpe, L.; Primo, L.; Cavalli, R. Prevailingly cationic agmatine-based amphoteric polyamidoamine as a nontoxic, nonhemolytic, and "stealthlike" DNA complexing agent and transfection promoter. *Biomacromolecules* **2007**, *8* (5), 1498-1504, http://dx.doi.org/10.1021/bm061126c.
4. Ferruti, P.; Mauro, N.; Falciola, L.; Pifferi, V.; Bartoli, C.; Gazzarri, M.; Chiellini, F.; Ranucci, E. Amphoteric, prevailingly cationic L-arginine polymers of poly(amidoamino acid) structure: synthesis, acid/base properties and preliminary cytocompatibility and cell-permeating characterizations. *Macromol. Biosci.* **2014**, *14* (3), 390-400, http://dx.doi.org/10.1002/mabi.201300387.
5. Marques, J.; Valle-Delgado, J.J.; Urbán, P.; Baró, E.; Prohens, R.; Mayor, A.; Cisteró, P.; Delves, M.; Sinden, R.E.; Grandfils, C.; de Paz, J.L.; García-Salcedo, J.A.; Fernàndez-Busquets, X. Adaptation of targeted nanocarriers to changing requirements in antimalarial drug delivery. *Nanomedicine: NBM* **2017**, *13*, 515-525, https://doi.org/10.1016/j.nano.2016.09.010.

© 2018 by the authors. Submitted for possible open access publication under the terms and conditions of the Creative Commons Attribution (CC BY) license (http://creativecommons.org/licenses/by/4.0/).
